# Eukaryotic cell size regulation and its implications for cellular function and dysfunction

**DOI:** 10.1152/physrev.00046.2023

**Published:** 2024-06-20

**Authors:** Yagya Chadha, Arohi Khurana, Kurt M. Schmoller

**Affiliations:** Institute of Functional Epigenetics, Molecular Targets and Therapeutics Center, Helmholtz Zentrum München, Neuherberg, Germany

**Keywords:** cell cycle, cell growth, cell size, organelle homeostasis, protein homeostasis

## Abstract

Depending on cell type, environmental inputs, and disease, the cells in the human body can have widely different sizes. In recent years, it has become clear that cell size is a major regulator of cell function. However, we are only beginning to understand how the optimization of cell function determines a given cell’s optimal size. Here, we review currently known size control strategies of eukaryotic cells and the intricate link of cell size to intracellular biomolecular scaling, organelle homeostasis, and cell cycle progression. We detail the cell size-dependent regulation of early development and the impact of cell size on cell differentiation. Given the importance of cell size for normal cellular physiology, cell size control must account for changing environmental conditions. We describe how cells sense environmental stimuli, such as nutrient availability, and accordingly adapt their size by regulating cell growth and cell cycle progression. Moreover, we discuss the correlation of pathological states with misregulation of cell size and how for a long time this was considered a downstream consequence of cellular dysfunction. We review newer studies that reveal a reversed causality, with misregulated cell size leading to pathophysiological phenotypes such as senescence and aging. In summary, we highlight the important roles of cell size in cellular function and dysfunction, which could have major implications for both diagnostics and treatment in the clinic.

## Graphical abstract

**Figure:**
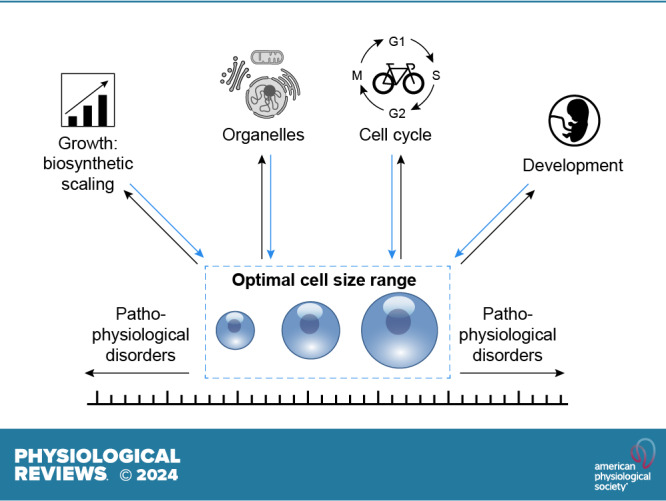

CLINICAL HIGHLIGHTS Cell size emerges as a major parameter controlling cell function. It is tightly linked to cell growth and cell cycle progression, dictates the size of many organelles, and through fundamental links to transcription and translation modulates the composition of the cellular proteome. While cell size alterations have been associated with many human diseases and aging, the causal relationships were long unclear. Recent studies revealed that increased cell size can lead to cellular malfunction and impaired cell cycle progression and thereby acts as a causal driver of cellular senescence and aging. This has broad consequences for diagnosis and potential therapeutic strategies, in particular for cancer treatment, where large cell size can be employed as a target for selective drug sensitivity. Future work now has to investigate the mechanisms through which cell size may impact diseases and further explore the potential of cell size as a diagnostic marker and predictive parameter for customized treatment and patient stratification.

## 1. INTRODUCTION

**FIGURE 1.**
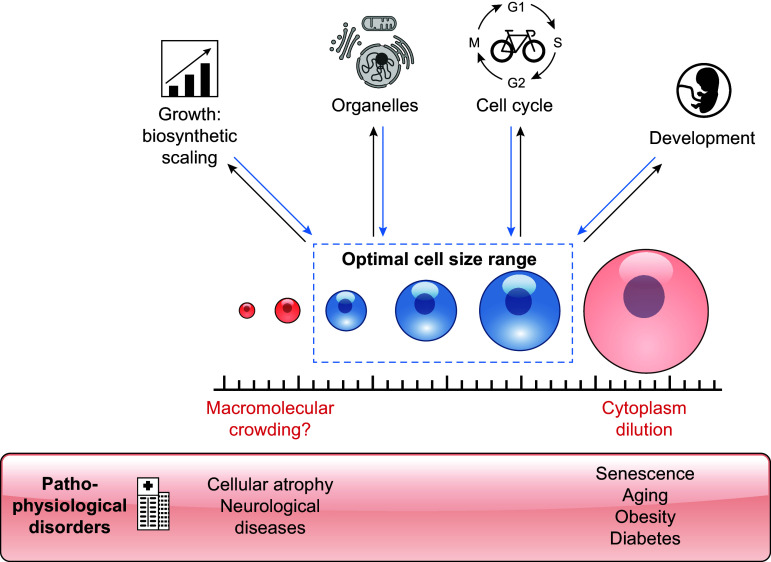
Cell size is tightly linked to biosynthesis, organelle homeostasis, cell cycle regulation, and developmental processes. Misregulation of cell size is detrimental to cell function and associated with diseases and aging.

Depending on ecological niche, cell type, and environment, the diameter of eukaryotic cells ranges from less than a micrometer to several centimeters. In other words, their volume spans more than 14 orders of magnitude. While cells of any size are built on the same fundamental biological processes, cell size has a major impact on cell function (FIGURE 1): it is a major determinant of biosynthetic capacity, setting the scale of transcription and protein production (1). The timescale of ideal passive diffusion across the cell increases with the diameter squared. Moreover, cell size governs intracellular organization, with many organelles increasing either in size or abundance with overall cell size (2), and the molecular composition of a cell changes with its size because not all cellular molecules increase in direct proportion to cell volume (3–5). Because cell size governs cell function, it is not surprising that proliferating cells control their size by balancing cell growth and cell division. To achieve this, they are able to sense their own size and use this information to modulate cell growth and cell cycle progression accordingly (6).

Changes in cell size are ubiquitous in biology. On one hand, regulation of cell function, for example, during development or in response to changing environments, typically leads to cell size adaptation. On the other hand, cellular dysfunction is often accompanied by altered cell size. For example, unusual and heterogeneous cell size is typical for cancer (7). Moreover, aging and cellular senescence are associated with an increase in cell size in yeast and mammals (8, 9). In principle, these changes could be downstream effects because cell size is sensitive to both cell growth and cell cycle progression. For example, the large size of senescent cells was long considered to be a consequence of cells still growing despite the permanent cell cycle arrest. However, recent studies revealed that increased cell size itself promotes senescence by disrupting cell function, reverting our understanding of the underlying causality (9).

**FIGURE 2.**
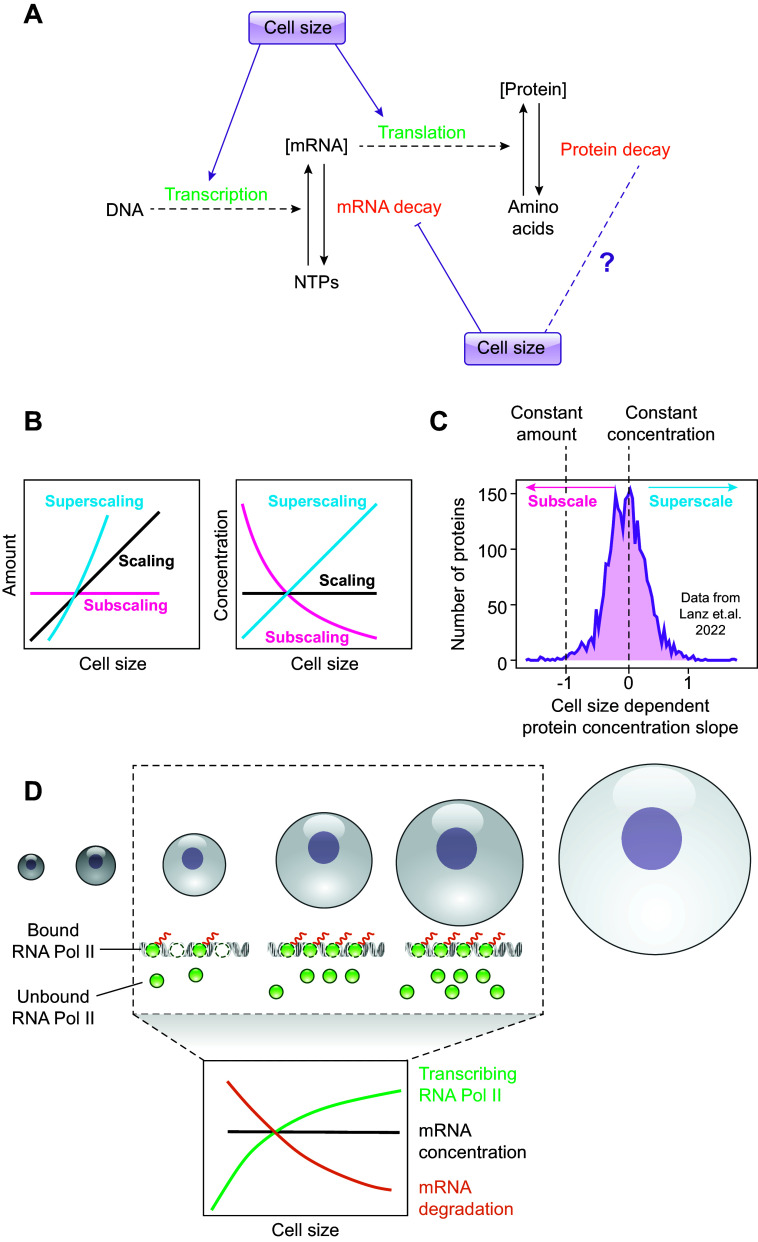
*A*: cell size can affect protein homeostasis at the steps of transcription, translation, and transcript or protein degradation. *B*: this can lead to scaling, subscaling, or superscaling behavior of individual transcripts or proteins. *C*: a broad range of scaling behavior is observed in human lung fibroblast cells (5). *D*: scaling of global transcript amounts in budding yeast is achieved through a combination of a cell size-dependent increase of transcription due to limiting polymerase II (Pol II), and a size-dependent decrease of mRNA degradation.

During the last decades, cell size was studied by a small group of researchers interested in understanding how cells control their size and how intracellular processes are coordinated with changes in cell size. Only recently, new insights into how changes in cell size cause a broad reorganization of the cellular transcriptome and proteome (10) have sparked attention (FIGURE 2) and have led to an increasing awareness that because cell size impacts virtually all intracellular processes, it must be considered in many biological contexts. Still, across cell biology, the role of cell size is often ignored.

Focusing on mononucleated cells, we here aim to give an overview of how cell size governs the function of eukaryotic cells, how cells can control their size, and how failure to maintain the correct size leads to cellular dysfunction and disease. Note that unless clarified otherwise, we use the term “cell size” to interchangeably refer to cell volume, cell mass, and cellular dry mass, all of which are typically, but not always, strongly correlated.

## 2. CELL SIZE GOVERNS BIOSYNTHESIS AND CELLULAR COMPOSITION

In essence, a cell is a sophisticated living machine that functions using an intricate network of biochemical reactions. The rate of these reactions is governed by the concentration of the molecules involved. If concentrations control biological function, for a cell to increase in size without compromising the efficiency of the intracellular processes, macromolecules must increase in amount with size to maintain their constant concentrations. Such a proportional increase with cell size, leading to constant concentrations, is often termed “scaling.” Alternatively, if the concentration decreases, that is the molecule is diluted with size, it is called “subscaling.” If the concentration increases, that is the molecule is concentrated with size, it is called “superscaling” (FIGURE 2*B*). Regulating these different scaling relationships allows the cell to adjust cell function according to the new physiological state associated with the changed cell size and account for the associated physical constraints. Emphasizing the importance of the correct scaling relationships, misregulation of size-dependent scaling in exceedingly large cells has been shown to be detrimental and recently has been proven to be a causal driver of senescence (3, 11) (see sect. 8).

Typically, total cellular transcript and protein amounts increase roughly proportionally with cell size, such that their concentration is maintained (1, 12–19). The absolute levels of transcripts and proteins depend on the rates of their synthesis and degradation, and in principle, both processes may be regulated with cell size (FIGURE 2*A*). Studies spanning across four decades show larger cells to have higher transcription (12, 14, 16, 20–25) and translation rates (20, 24–27). The relationship of global mRNA and protein degradation rates with cell size is less clear, but early evidence indicated that it plays a less prominent role in achieving the proportional scaling (16, 24, 26, 28). However, as discussed in more detail below, recent evidence in budding yeast demonstrates that at least for mRNA, degradation rates decrease with cell size, which especially for large cells is necessary to maintain constant mRNA concentrations (29) (FIGURE 2*D*).

### 2.1. Limiting Polymerase and Degradation Couple mRNA Amounts to Cell Size

Total transcript amounts scale with cell size at least in part because transcription rate increases with size. This raises the question of how the transcription rate increases with cell size even if the genomic DNA content, which is the template for transcription, remains constant. The observation that the cellular content, not just the genome, determines the transcriptional output, together with the fact that components of the transcriptional machinery also scale with cell size, led to the hypothesis that the transcription rate is limited by a component of the transcriptional machinery (1, 14, 16, 30–32). Consistent with this idea, the scaling of transcription rate breaks down at large cell sizes (29) because at this point, the DNA template for transcription, i.e., the genes, becomes limiting. Accordingly, in this cell size regime, the transcription rate per cell can be further increased by an increase of ploidy.

One obvious candidate for the limiting factor coupling transcription to cell size is the polymerase itself, because RNA polymerase II (Pol II) abundance increases with cell size across organisms (16, 19, 29, 32) and its genome occupancy has been shown to increase with cell size in budding and fission yeast (29, 32). Indeed, Swaffer et al. (29) recently established Pol II as the key limiting factor in budding yeast by manipulating the concentration of Pol II subunits. Specifically, consistent with the haploinsufficiency of RNA Pol II genes in yeast (33), they show that reducing the amount of the catalytic RNA Pol II subunit Rpb1 (encoded by POLR2A in humans) proportionally decreased the RNA Pol II bound to the genome, strongly suggesting that the transcription rate also decreases accordingly. Similarly, overexpressing RNA Pol II subunits was sufficient to increase the RNA Pol II genome occupancy (29). Importantly, in contrast to a simple model where almost all RNA Pol II is bound to the genome, Swaffer et al. (29) demonstrated that only about half of the RNA Pol II is loaded onto the genome, and, as a consequence of mass action kinetics, its loading to DNA increases sublinearly with size. To compensate for the sublinear cell size scaling of the transcription rate, the mRNA degradation rate decreases with cell size to maintain constant concentrations of total transcript (FIGURE 2*D*).

In summary, while the proportional increase of Pol II amounts with cell size explains an increase in transcription rates, it also becomes clear that both transcription and degradation contribute to the global scaling of mRNA amounts with cell size. Since mRNA degradation and transcription are coupled via feedback mechanisms in yeast and humans (19, 34–36), it seems likely that in addition to the limiting-machinery mechanisms, additional layers of regulation are in place to ensure robust scaling of transcript amounts with cell size.

Akin to total mRNA, total rRNA (18S, 26S, and 5S) and tRNA (4S) amounts per cell also roughly scale with cell size in budding and fission yeast (23, 37, 38). The amount of rRNA and tRNA per cell as well as the promoter occupancy of RNA Pol III also increases during the hypertrophic growth of cardiomyocytes (39), indicating that this trend may also hold true for animal cells. The scaling behavior of other noncoding RNAs is largely unknown. Furthermore, whether RNA Pol I and RNA Pol III limit the transcription of their respective transcripts and thereby control their size scaling remains to be investigated.

### 2.2. Translational Capacity Increases with Cell Size

Since both mRNA and ribosome amounts (4, 5, 23, 40, 41) increase with cell size, it seems intuitive that a simple model where ribosomes are limiting for global translation accounts for the size dependence of total protein amount. However, recent evidence suggests that while the global translation rate per cell increases with size, this scaling is sublinear, especially in large cells (25, 42). Besides a nonperfect scaling of ribosome amounts, one explanation could be that the fraction of active ribosomes (43, 44) controls total translation and decreases with cell size. Interestingly, such a cell size-dependent decrease of translation rate is not observed when comparing cells of different ploidy and thus different sizes. Instead, increasing ploidy leads to an increase in cellular translation rate at a given cell size such that linear size scaling is maintained between cells of different ploidy (42). However, the exact dependence of protein amounts on ploidy and cell size might differ between species, as it has been reported that even when comparing yeast strains with different ploidy, protein amounts subscale with cell volume (45).

### 2.3. Individual Proteins Are Differentially Regulated with Cell Size

Even though total transcript and total protein are maintained at roughly constant concentrations within the physiological cell size range, recent studies have shown that at the level of individual genes, the concentration of different proteins and transcripts can change with cell size (5, 41, 46). A prominent example of a class of proteins that decreases in concentration is histones, which need to be maintained at a constant histone-to-DNA stoichiometry (4, 5, 41, 47–49). Similarly, cell cycle inhibitors such as Whi5 in yeast (4, 50–53), Rb in mammals (54–56), and KRP4 in *Arabidopsis thaliana* (*57*) subscale, leading to their dilution in bigger cells. In contrast, the fission yeast cell cycle activators Cdc25 and Cdc13 superscale, leading to higher concentration in larger cells (58–61). As discussed in more detail in sect. 7, this differential size dependence of cell cycle regulators can serve as the basis for cell size regulation.

Recent studies have conducted transcriptomics and mass spectrometry to reveal the genome-wide size scaling of transcripts and proteins in human cell lines as well as in budding yeast by sorting cells based on their size or by manipulating their cell size (4, 5, 41, 42, 62). A key finding of those studies is that individual genes show a wide distribution of scaling behaviors, ranging from histones and other DNA binding proteins on the subscaling end of the spectrum to mitochondrial and metabolic proteins involved, for example, in TCA cycle and glycolysis on the superscaling end (FIGURE 2*C*). Interestingly, at least the global patterns of scaling show high similarity between different human cell types (5, 46) and budding yeast (41). Moreover, the proteome composition of large cells resembles characteristics associated with senescence in mammals (5, 46) and starvation-like conditions in budding yeast (41). This suggests that DNA becomes limiting for transcription in large cells, leading to inadequate biosynthetic scaling (3) that ultimately drives cells toward senescence and a starvation-like phenotype.

Given the broad distribution of scaling behaviors between individual genes, the question arises of how this differential scaling is established. At least for some genes, including histones and the cell cycle inhibitor Whi5 in budding yeast, subscaling is established at the transcript level (4, 48, 58), and the promoter alone can mediate this behavior (4, 48). One potential mechanism for such subscaling is that for those specific genes, the DNA template rather than Pol II is limiting for transcription already in the physiological cell size range (31, 48, 63, 64). However, transcription is not the only step of protein homeostasis at which cell size dependence can be controlled. It was recently found that for the mammalian cell cycle inhibitor Rb, protein degradation controlled by the E3 ligase UBR5 is necessary for the decrease of its concentration associated with growth in G_1_ (56).

Across genes, subcellular localization has been identified to be the strongest predictor of the scaling behavior of proteins in human cells, while mRNA size scaling is the strongest predictor in the case of budding yeast. In both cases, using multiple parameters including mRNA scaling slope, protein turnover, codon affinity score, and subcellular localization improved the prediction significantly, indicating that protein scaling at the individual gene level is regulated both transcriptionally and posttranscriptionally (5, 41).

### 2.4. Cell Size Dependence of Other Macromolecules and Metabolic Scaling

Given the plethora of tools and techniques available for the quantification of proteins and nucleic acids, it is not surprising that these macromolecules are the best studied in the context of cell size, while the cell size scaling of lipids and polysaccharides is less clear. From early studies, we know that larger human fat cells have a higher rate of lipid synthesis in vitro (65), and fat deposits scale linearly with adipocyte size in migratory birds (66) as well as rats (67). Additionally, total cellular phospholipids, the building material for cell membranes, correlated well with cell size, while cholesterol synthesis peaked at a certain cell size in mouse fibroblast cells (68). A recent multi-omics-based study quantified RNAs, proteins, metabolites, and lipids for budding yeast cells that were cell cycle synchronized by elutriation and separated by size and used as a proxy for the cell cycle stage. The relative composition of the cellular metabolome changed during the cell cycle; however, the absolute metabolite concentrations and the causal contribution of cell size and cell cycle remain unclear (69). Since macromolecule biosynthesis requires the right amounts of the appropriate monomers, such as amino acids, nucleotides, nucleosides, fatty acids, or simple sugars, it would be interesting to see if and how the concentration of these metabolites changes with cell size.

Besides the cell size dependence of various macromolecules and metabolites, the link between metabolic activity and cell size is also still poorly understood. Allometric scaling relationships similar to Kleiber’s law, which describes a sublinear power-law scaling of an animal’s metabolic rate with its body mass, have also been observed for individual cells. As reviewed elsewhere (70, 71), we are only at the beginning of revealing the origin of these scaling laws and, in particular, their intricate link to multicellularity, tissue context, and organism size (72).

### 2.5. Cell Density is Modulated by Cell Size

Cell density is a property determined by the cumulative concentrations of all cellular components, and cell density homeostasis therefore depends on a tight coordination of biosynthesis and cell growth (73, 74). Depending on the experimental approach, the ratio of dry mass to total cell volume (cellular mass density) (75, 76) or dry mass to dry volume (77) can be used as proxy measurements. Dry mass is dominated by large macromolecules, including proteins, nucleic acids, and lipids. Cell volume on the other hand is dominated by highly abundant small metabolites such as ions and amino acids (78, 79). Reflecting its importance in cell function maintenance, cellular mass density is tightly regulated, even compared to dry mass or cell volume (76, 80) [see Neurohr and Amon (81) for a dedicated review]. However, it changes as a function of cell cycle (77, 82), cell differentiation, environmental conditions, diseases, and senescence (81, 83).

Since the individual concentration of many cellular components changes with cell size, in particular in large cells, cell density will change accordingly. Indeed, a drastic decrease in cell density has been observed in excessively large cells (3). Besides measurements of individual molecules, direct measurements of cell density will be needed to obtain a better understanding of its link to cell size. A prominent technique that uses suspended microchannel resonators (SMRs) to quantify cell density can measure the buoyant mass of the cell with high accuracy and sensitivity but does not provide spatial information (75, 80, 84, 85). By contrast, optical methods such as quantitative phase imaging or cryoelectron microscopy can also provide subcellular information (86–89). While all these techniques cannot differentiate between macromolecules and thus cannot be used to reveal the dry mass composition, it is now possible to distinguish various macromolecules as well as obtain their spatial information using Raman scattering (SRS) microscopy (83, 90, 91). For example, using quantitative Raman microscopy, Oh et al. (83) could resolve the contribution of protein and lipid concentrations to cytoplasmic dilution in senescent mammalian cells.

### 2.6. The Relationship Between Cell Size and Nuclear DNA

One of the most striking but still poorly understood correlations with cell size is that of genome content. Across species, and over a range of several orders of magnitude, eukaryotic cell size increases almost in proportion to genome size, which in this context is often referred to as the C value (92). By contrast, and highlighting the fundamentally different intracellular organization of DNA, a much weaker scaling is observed for bacteria (93). In addition, for cells of the same or of closely related species, including plants (94), yeast (95), frogs (72, 96), and human stem cells (97), cell size is often tightly linked to DNA content, leading to a proportional increase of cell size with cell ploidy. For example, in humans, polyploidy associated with increased cell size occurs in specific cell types, including megakaryocytes (98), cardiomyocytes (99), and hepatocytes (100), in particular during aging (101).

Interestingly, on an organismal level, polyploidy is often, but not always, also accompanied by an increase in body size. For example, many plants, particularly cultivated ones, are polyploid, with increased cell and body size (102). Similarly, the well-studied allotetraploid frog *Xenopus laevis* not only has larger cells than its diploid relative *Xenopus tropicalis* but also exhibits a larger body size. However, this relationship is not universal, since the dodecaploid *Xenopus longpipes* is smaller than *X. laevis*, while still having larger cells (96). Similarly, salamanders of different ploidy were found to have constant organism size despite scaling cell and nuclear sizes (103).

The correlations observed between genome content, nuclear size (see sect. 3.1), and cell size naturally suggest a mechanistic link. Two possible explanations are evident: first, nuclear size may be set by genome content and then as a consequence cause cell size to follow accordingly. However, recent progress in understanding nuclear size homeostasis suggests that nuclear volume is determined by osmotic pressure rather than the volume occupied by DNA. Consistent with this idea, nuclear size at a given cell size is rather independent of ploidy in yeast (104). Thus, even though it is clear that DNA content at least sets a lower limit of physically possible nuclear volumes, this is likely not the dominant reason for the correlation between cell size and genome content.

This leaves us with the second explanation, namely that increased DNA content supports larger cell size through its limiting effect on biosynthesis. As discussed in sect. 2.3, at large cell sizes, the template DNA becomes limiting for transcription (3, 14, 29) and thus ultimately for protein synthesis. Increasing cell ploidy then allows cells to overcome the problem of limiting DNA and enables larger cell sizes (105). Similarly, coevolution of increased genome size might therefore help to sustain larger cell size.

However, such a link between increased genome size and larger cell size requires that the increased genome size goes along with an increased amount of transcribed DNA, and matters are therefore complicated by the rather imperfect correlation between gene number and genome size across species (106). Moreover, it is important to note that even the relationship between cell size and ploidy is not universal, even for a given species. For example, endoreplication is common in plants and often leads to an increase in cell size. Still, the dependency of cell size on ploidy is tissue-specific in *A. thaliana* (107). Similarly, comparing the cell sizes of *Xenopus* species with different ploidy throughout development revealed that in the early embryo, cell size is determined by the egg size, while the size of the nucleus is dependent on the genome content. Only during development, a constant nuclear-to-cytoplasmic ratio is established, such that in adults, cell size then correlates with ploidy (96).

## 3. ORGANELLE SCALING WITH CELL SIZE

**FIGURE 3.**
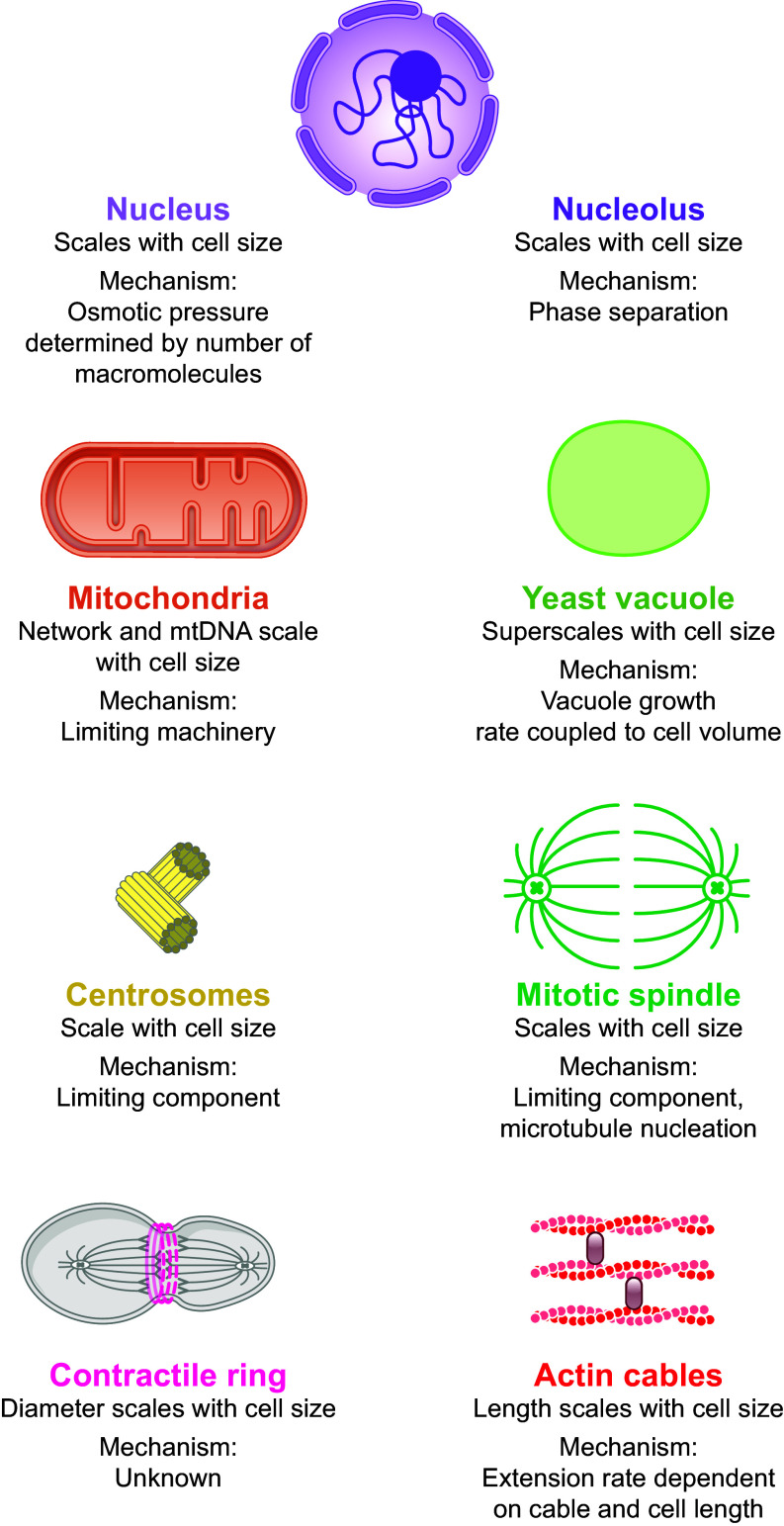
The size and/or number of many organelles is coupled to cell size. This is achieved through a wide range of mechanisms and can be regulated according to biological context.

As cells change their size, they also need to adjust the scale of intracellular structures and compartments accordingly. Across species, the emerging picture is that the size or abundance of many organelles, including the nucleus, mitochondria, and the mitotic spindle, increases roughly in proportion to cell volume (FIGURE 3). This leads to the question of how cells can couple organelle homeostasis to overall cell size. One intriguing regulatory principle is that organelle size could be set by a pool of limiting building blocks (108, 109). Since the global biosynthetic capacity of the cell increases with cell size (sect. 2), the abundance of those building blocks, for example, proteins, would then also increase with cell size. As a consequence, this would allow larger cells to build larger organelles. Conceptually related mechanisms rely on enzymes that are limiting for organelle maintenance and increase in abundance with cell size (110) or couple organelle formation to protein concentration and cell volume through phase separation mechanisms (111). While regulation of organelle homeostasis through limiting components provides an intuitive concept of how a coupling of organelle size to cell size can in principle be achieved, extensive studies on many different organelles and biological systems revealed a much more complex picture. First, different organelles are regulated by cell size through different mechanisms. Second, the regulation of a specific organelle can also vary depending on the biological context, including the species and its developmental stage. In the following, we will discuss the size dependence of major organelles and our current understanding of the underlying mechanisms. Additional information can be found in dedicated reviews (2, 112).

### 3.1. Nucleus

Maybe the most obvious relationship between organelle and cell size, noted already more than 100 years ago (113), is the constant ratio between nuclear and cell volume, leading to a constant nuclear-to-cytoplasmic volume ratio. Since across eukaryotes the volumes of both the nucleus and the cell itself scale with genome size (sect. 2.6), this suggested that nuclear DNA content sets the range of possible nuclear volumes (93). However, detailed measurements of nuclear sizes during the cell cycle revealed that in yeast nuclear volume increases with cell volume even at a given cell cycle stage and thus at a constant DNA content (104, 114). In addition, replication of DNA during the S phase does not lead to a dramatic increase in nuclear volume, suggesting that nuclear volume must be linked directly to cell volume rather than just to DNA content. More recently, attention has been brought to specific situations, where the coupling of nuclear and cellular size breaks down (115). It was then proposed that, instead, it is actually the nuclear-cytoplasmic density ratio that is maintained constant (116).

Therefore, how is nuclear size controlled? Analysis of fission yeast nuclear size homeostasis upon perturbation of the nuclear-to-cytoplasmic volume ratio revealed fast recovery on the time scale of the cell doubling time (117). This work led Cantwell and Nurse (117) to suggest that nuclear size is determined by the amount of nuclear content, which itself is given by the balance of nuclear import and export. This idea was developed into a refined model by two studies (118, 119), which proposed that the dominant pressure controlling nuclear volume is osmotic and the nuclear-to-cytoplasmic volume ratio is therefore determined by the number of macromolecules, that is proteins and RNA, in the nucleoplasm and cytoplasm. This model predicts a constant nuclear-to-cytoplasmic volume ratio across changing cell sizes. It also implies that the nuclear volume occupied by DNA is negligible for overall nuclear size and that the available nuclear membrane area is not a limiting factor. Recently, Rollin et al. (78) pointed out that in addition to proteins and RNA, the large pool of metabolites also plays a role in nuclear size homeostasis by contributing to nuclear and cell volume and diluting chromatin charge.

While this model provides an intriguing framework for nuclear size control, additional factors are likely to contribute, at least for some cell types and environments. Specifically, the increased nuclear-to-cytoplasmic ratio observed for small epithelial cells points toward a minimal nuclear size set by the volume occupied by chromatin (120). Along those lines, Biswas et al. (116) suggested that the entropic pressure exerted by chromatin contributes significantly (∼20%) to the volume of *X. laevis* nuclei. In addition, chromatin has an indirect effect on nuclear size homeostasis by regulating nuclear import. In mammalian cells, an additional contribution may come from forces exerted on the nuclear envelope by the cytoskeleton. By modulating nuclear transport, mechanical forces can then lead to a decoupling of nuclear and cell volume during cell growth (121).

### 3.2. Nucleolus

Besides the nucleus itself, the size of the nucleolus also scales with cell size. Using *Caenorhabditis elegans* development as a model, Weber and Brangwynne (122) showed that nucleolar scaling can be explained by a phase separation mechanism of its components. During development, this leads to a proportional increase of nucleolar volume with cell volume, even though nuclear volume subscales (123). Highlighting the importance of nucleolar size regulation, and consistent with its role in ribosome biogenesis, organismal growth rate depends on the size of the nucleolus relative to the cell.

### 3.3. Mitochondria and Chloroplasts

Mitochondria and chloroplasts are thought to have originated from the endosymbiotic uptake of an alphaproteobacterium and a cyanobacterium, respectively. They both still maintain their own genome, which often is present in multiple copies. Accordingly, proliferating cells need to coordinate the growth of mitochondria and chloroplasts, as well as replication of their respective DNA with cell growth and division. One intuitive strategy to maintain stable concentrations of an endosymbiont is for the host cell to control endosymbiont division and directly couple it to its own cell cycle. Indeed, tight coupling to the host cell cycle has been observed for the division of the endosymbiont of the trypanosomatid *Angonomas deanei* (124) and chromatophores of *Paulinella chromatophora* (125). The latter are the only photosynthetic organelles that originated from a primary endosymbiotic event separate from the origin of chloroplasts. For some species, including the kinetoplastid *Trypanosoma brucei* (126), cell cycle-dependent mitochondrial DNA (mtDNA) replication and mitochondria division are used as strategies for mitochondrial homeostasis.

For many eukaryotes, however, mitochondrial fission and mtDNA replication can occur throughout the cell cycle, allowing cells to regulate the amount of mitochondria depending on cell type and external cues such as nutrient availability. To still achieve stable mitochondrial homeostasis, coupling mitochondrial biogenesis to cell growth provides an alternative strategy to regulation by the host cell cycle. It has been shown in budding yeast (110, 127), insect (128), and mammalian cells (129, 130) that for a given cell type and condition, the mitochondrial network amount increases with cell size. At the same time, in budding yeast, the mtDNA copy number also increases in proportion with cell volume, which has been attributed to nuclear-encoded limiting machinery, in particular the mtDNA polymerase Mip1 (homolog of human POLG) and the mtDNA binding protein Abf2 (homolog of TFAM), whose abundance increases with cell volume (110). Thus the mechanism underlying the coupling of mtDNA to cell volume is reminiscent of increasing amounts of limiting Pol II leading to a global increase of transcription in larger cells (29) (sect. 2.1).

Although mtDNA is a major regulator of mitochondrial function, cell volume impacts mitochondria through additional pathways. First, in budding yeast, the mitochondrial network and mtDNA are coupled to cell volume through independent mechanisms, since the mitochondrial network scales with cell volume even in mutant cells that lack mtDNA, and a decreased mitochondrial network amount does not necessarily cause a decrease in mtDNA (110). Second, despite scaling amounts of mitochondria, the metabolic function of animal cells varies across cell volumes. In particular, cells show maximal membrane potential and oxygen consumption at intermediate cell volume, potentially contributing to the optimal cell volume range (128, 131).

Similar to the coordination of mitochondria and cell growth observed from yeast to mammals, various plants have been reported to coordinate chloroplast homeostasis with cell size. Among others, chloroplast number was observed to increase with the cell size of wheat (132) and *A. thaliana* mesophyll cells (133). In addition, chloroplast number and total chloroplast area increase with cell face area of spinach mesophyll cells (134). Finally, chloroplast DNA copy numbers correlate with cell size during the *Chlamydomonas reinhardtii* cell cycle (135).

Pointing toward fundamental constraints leading to size scaling of organelles that emerged from metabolic endosymbiosis, “nitroplasts” (136), nitrogen-fixing endosymbiotic cyanobacteria, also increase in size with the size of their host, the alga *Braarudosphaera bigelowii* (137).

### 3.4. Vacuoles

Both the surface area and the volume of budding yeast vacuoles increase with cell volume. The exact scaling relationships may vary between different strains, but in any case, vacuole volume was observed to superscale with cell volume, meaning that an increase in cell volume leads to a more than proportional increase in vacuole volume (138). Vacuole size scaling can be explained by the relative growth rates of the vacuole and the cell, with no need for active feedback adjusting vacuole growth rate to vacuole size (139).

### 3.5. Centrosome

The scaling of centrosome volume with cell volume observed during *C. elegans* (140) and zebrafish (141) early development is a prominent example for which a simple “limiting component” model has been proposed. In particular, Decker et al. (140) identified the pericentriolar material protein SPD-2 to be a limiting factor.

### 3.6. Mitotic Spindle

Cytoskeletal structures built from biopolymers such as microtubules or actin are another category of cellular subunits that needs to be regulated with changing cell size. Probably the best-studied example is the mitotic spindle, which has been shown to scale with cell volume in multiple species. By analyzing spindles formed in *X. laevis* cell extract confined in vitro into defined volumes, Hazel et al. (142) and Good et al. (143) could demonstrate that spindle size is determined by a limiting cytoplasmic component. One candidate for such a limiting protein is the microtubule polymerase XMAP215 (144, 145), which would be consistent with the cell size-dependent change of spindle microtubule growth rate (146). However, complicating matters, careful analysis of spindles in zebrafish embryos revealed that while at small sizes, microtubule growth rate increases with cell size, growth rate is constant at intermediate cell sizes, where spindle size still increases. Rieckhoff et al. (147) found that in this intermediate regime, the number of microtubules in the spindle increases. They propose that spindle size is determined by microtubule nucleation. A nucleation inhibitor, which is partitioned to the cell membrane, then links the scaling relationship to the cell surface area. At small cell sizes, this leads to polymerization becoming limiting. This model is also consistent with the finding that *X. laevis* extracts from different developmental stages form differently sized spindles because importin-α, an inhibitor of the microtubule destabilizing factor kif2a, is partitioned to the membrane, leading to lower concentrations at later developmental stages (148).

### 3.7. Contractile Ring

Another cytoskeletal organelle that needs to be coordinated with cell size is the contractile actomyosin ring. Naturally, in organisms with symmetrically dividing cells, such as *C. elegans*, the actomyosin ring is larger in bigger cells. Nevertheless, a scaling of constriction rate and initial ring size allows cells to complete cytokinesis within a cell size-independent time period (149). Interestingly, even the diameters of the actomyosin and septin ring at the bud necks connecting budding yeast buds with their mother cells scale roughly with the diameter of the mother cell (150, 151). While not strictly coupled, the cell size dependence of these structures is likely intertwined with the formation of the Cdc42 polarization cluster that recruits the septin ring to the presumptive bud site (151). Already the Cdc42 cluster scales in size with the cell size, which, in contrast to fission yeast (152), in budding yeast is not due to the change in local radius of curvature.

### 3.8. Actin Cables

Finally, in budding yeast, actin cables were shown to scale in length with cell length. Rather than through a limiting-pool mechanism (153), this has been proposed to be achieved through a cable extension rate that decreases with cable length, in a manner that is dependent on cell length (154).

## 4. OPTIMAL CELL SIZE

Cell size has a profound impact on macromolecular (sect. 2) and organellar composition of the cell (sect. 3). In addition, it is linked to metabolic functions. For example, carbon fixation, respiration, as well as nutrient uptake and content have been observed to increase with cell size across plankton species (155–157). Moreover, relative photosynthesis and growth rates as well as mitochondrial efficiency peak at intermediate-sized cells and gradually decrease in smaller or larger cells (128, 155, 156). It is therefore not surprising that cells intrinsically maintain their size within a narrow range, which we here refer to as the “optimal” cell size range. However, the optimal size range drastically varies across cell types, species, and environmental conditions. To date, what factors dictate the varying optimal cell size across different taxa and cell types remains an enigma. However, due to the different size ranges and different biological functions, it seems unlikely that there is a single universal determining factor. For example, a recent analysis of thermal acclimation of phytoplankton suggests that cell size is set by a delicate balance between nutrient uptake, lipid dynamics, protein synthesis, and more (158).

In both unicellular and multicellular organisms, recent evidence indicates that relative growth rate, that is growth rate per unit mass, peaks at intermediate cell sizes (159–164). The decline of growth rate in exceedingly large cells has been attributed to DNA becoming limiting and consequential cytoplasm dilution (3, 14). By contrast, it is far less understood what happens when cells are too small (161).

In unicellular organisms, achieving maximal growth and proliferation rate is thought to be a major determinant of the optimal cell size range. Work on resource allocation in bacteria suggests that maximization of growth rate can explain the adaptation of optimal cell size, for example, to different nutrients (165, 166) or upon overexpression of useless protein (165, 167). In rich nutrients, a larger proteome fraction is dedicated to biosynthesis, including ribosomes, which requires larger cell sizes (168).

**FIGURE 4.**
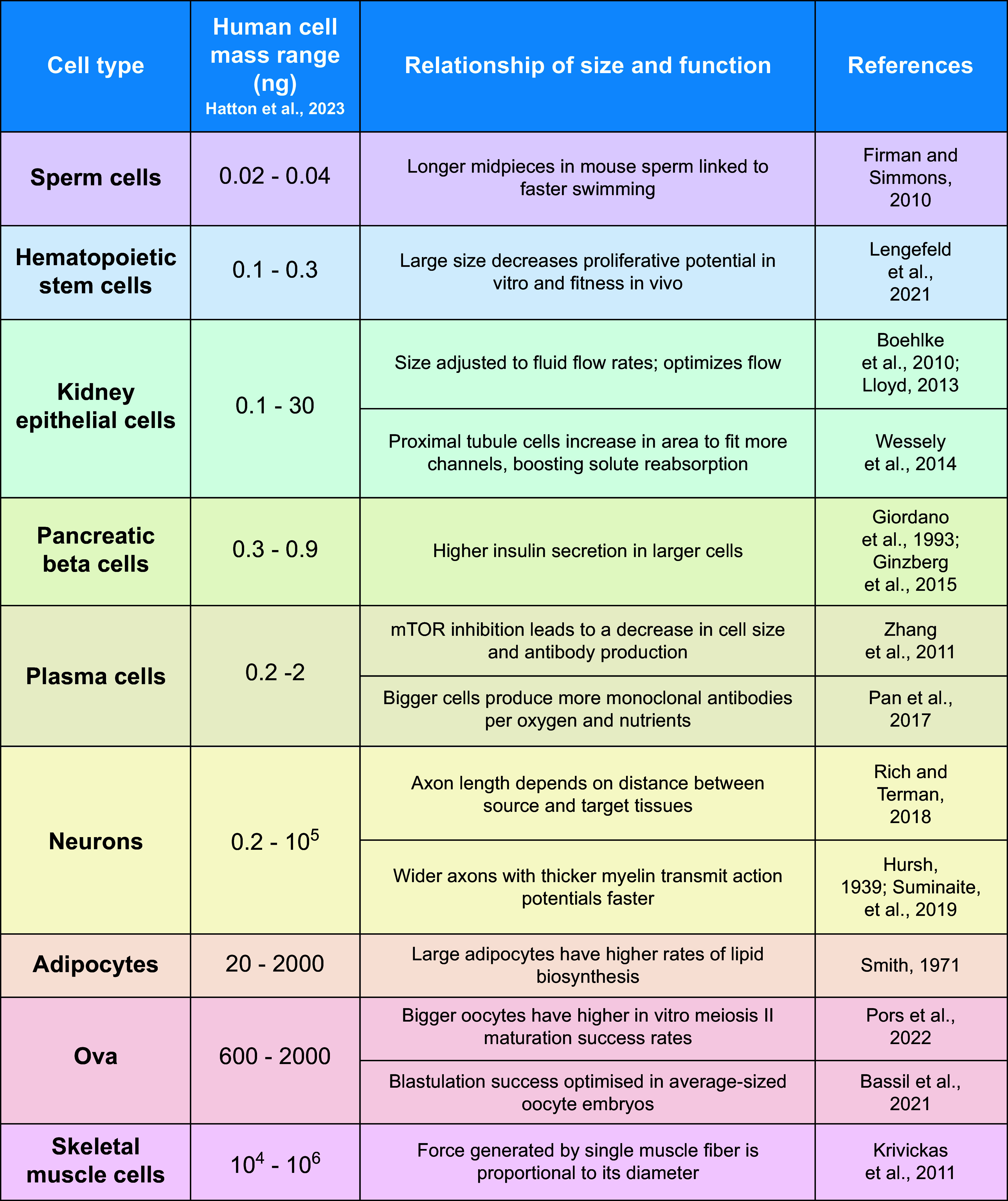
In mammals, cell size varies across different cell types (169), potentially to aid specific cell functions. Cell types where cell size has been correlated with cell function include sperm (170), hematopoietic stem cells (11), kidney epithelial cells (171–173), pancreatic beta cells (7, 174), plasma cells (175, 176), neurons (177–179), adipocytes (65), ova (180, 181), and muscle cells (182).

In multicellular species, cell-type specific function is also considered to be a significant factor in determining optimal cell size (161) (FIGURE 4). For example, certain neurons maintain longer axon lengths to physically conduct electrical signals over longer distances toward target tissues, whereas interneurons have shorter axon lengths since they connect over shorter distances (177). Mature oocytes, the biggest mononucleated human cells, contain a huge number of maternal factors deposited during oogenesis that are essential for embryonic development. Indeed, oocyte size is considered a good biomarker for selecting oocytes for in vitro fertilization (180, 181). Another level of complexity in multicellular organisms arises from the need for organ homeostasis. To maintain organ functionality, alterations in cell number can be compensated by cell size (183–185) and vice versa (103).

One additional noteworthy example where cell size seems to be linked to a specific cell function is stem cells. Stem cells are undifferentiated cells that have a self-renewal property, as well as the ability to differentiate into a plethora of different cell types with specialized functions and morphological characteristics, including cell size. Many studies across different multicellular species found that one of the most common features among stem cells is their small size (186, 187). Moreover, the criteria used for the identification of human induced pluripotent stem cells (iPSCs) not only include expression of pluripotency factors but also morphological features, in particular a small cell size (188). Linking small cell size to stem cell function, Lengefeld et al. (11) showed that the stem cell renewal potential of enlarged hematopoietic stem cells (HSCs) was negatively impacted. This is due to a decrease in their ability to proliferate rather than a decreased differentiation potential and was shown for both naturally large HSCs and HSCs whose size was increased by drug treatment. However, more generally the small size of stem cells also raises the question of how much of a role cell size plays in controlling cell fate decisions during development and differentiation.

## 5. CELL SIZE AS A REGULATOR FOR DEVELOPMENT AND DIFFERENTIATION

**FIGURE 5.**
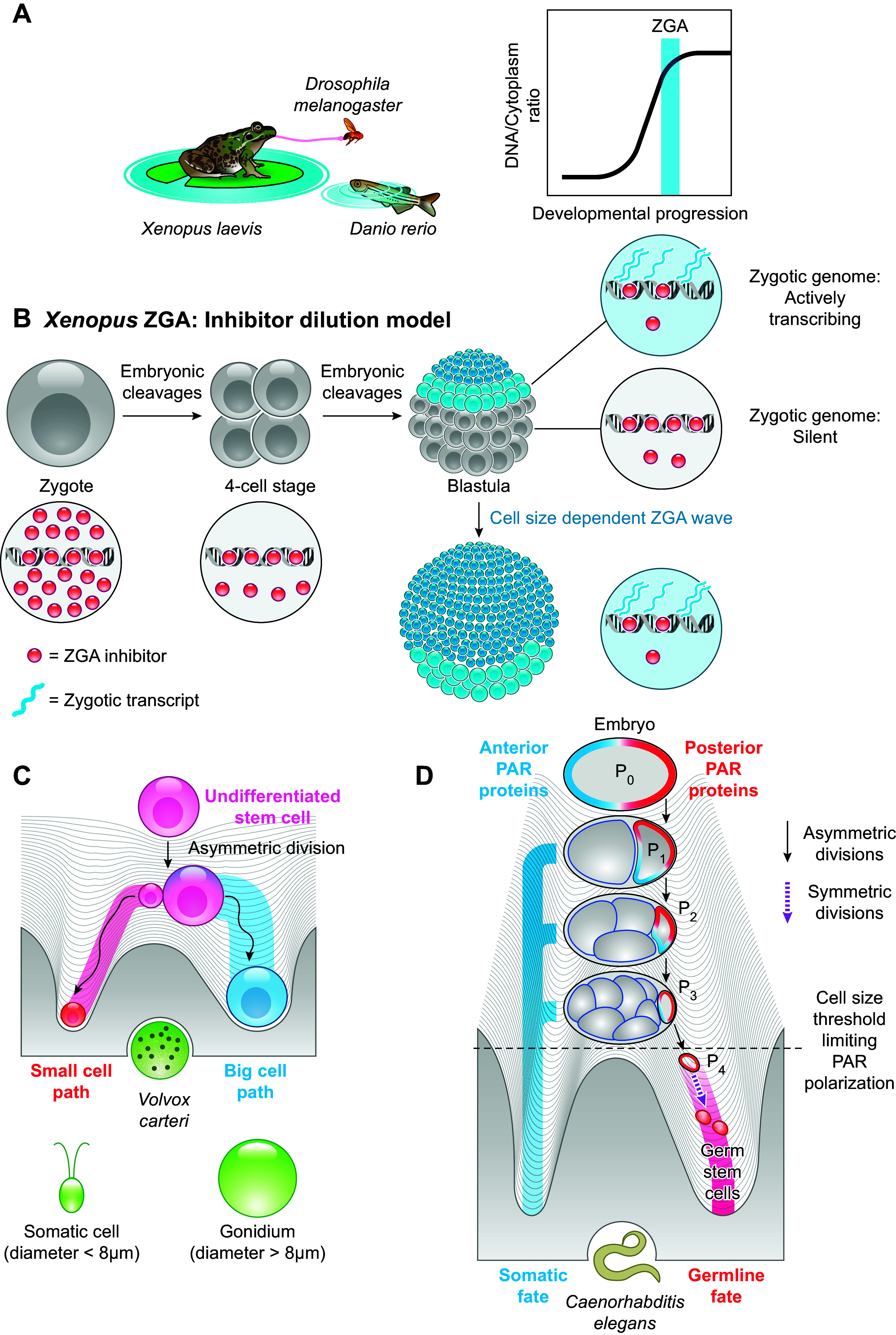
Cell size acts as a regulator of embryonic development and differentiation. *A*: in fish, fly, and frog embryos, zygotic genome activation (ZGA) and midblastula transition (MBT) depend on an increasing DNA-to-cytoplasm ratio. *B*: during the multiple cleavage divisions, repressors of ZGA and MBT are titrated against exponentially increasing DNA concentrations. Consequently, the spatial gradient of cell size in *Xenopus* embryos leads to a dependence of ZGA timing on cell position in the embryo. *C*: cell size is linked to cell fate specification in *Volvox carteri* embryos. *D*: asymmetric divisions in *Caenorhabditis elegans* embryos lead to a successive decrease in cell size and a change in the polarization of PAR proteins in the P lineage. When cell size decreases below a certain threshold, PAR polarization is disrupted, which triggers symmetric divisions and germline fate specification.

Development of a multicellular organism encompasses cells differentiating into various cell types and organizing into tissues, organs, and organ systems. During differentiation and various stages of development, cell size can change drastically. While in part this can be understood as a downstream consequence of producing optimally sized cell types, cell size can also act as a regulator of developmental processes (FIGURE 5).

### 5.1. DNA-to-Cytoplasm Ratio Controls Zygotic Genome Activation

Early animal development starts from an extremely large totipotent single cell, the zygote, that undergoes successive cleavage divisions, leading to an exponential decrease in cell size and consequently an increase in the DNA-to-cytoplasm ratio. After a fixed number of cleavage divisions, which varies across species, the embryo then transitions from rapid synchronous cleavages to slower nonsynchronous mitotic divisions. This stage is called midblastula transition. The initially transcriptionally inactive embryonic genome also transitions to an active state, a process that is termed zygotic genome activation (ZGA).

Several studies indicate that ZGA timing correlates with a threshold DNA-to-cytoplasm (or nucleus-to-cytoplasm) ratio in *Drosophila* (189–191), *Xenopus* (192–195), and zebrafish (196, 197) (FIGURE 5*A*). The mechanism behind this correlation involves the titration of maternally deposited histones against constant DNA amounts during early embryonic cleavages (195, 198–200). Moreover, the titration of replication factors (201) and the competition between histones and transcription factors for DNA binding (198) have also been shown to play a role in ZGA timing in *Xenopus* and zebrafish embryos, respectively. In addition, *Xenopus* embryos have a cell size-specific spatial distribution of blastomeres, where small cells are located at the animal pole while big cells are located on the vegetal pole. ZGA is first triggered in the small-sized cells, and then this activation wave gradually flows toward the other pole in a cell size-dependent manner (202) (FIGURE 5*B*).

### 5.2. Cell Size Impacts Cellular Differentiation

In addition to its importance for ZGA in early embryos, cell size also has been linked to cell differentiation. For example, an increase in the size of *Arabidopsis* root meristems is essential for initiating differentiation and consequently for root development (203). Moreover, the fate of murine bone marrow mesenchymal stem cells (mMSCs) can be manipulated by cell volume changes induced through osmotic perturbations (204). In hypoosmotic conditions, mMSCs increased in volume and differentiated into adipocytes. By contrast, in hyperosmotic conditions, mMSCs decreased in volume and differentiated into osteocytes. However, it is still unclear to what extent this is explained by cell volume-dependent density changes or other downstream effects of osmotic stress.

While correct regulation of stem cell size is important for differentiation, cell size can also act as a regulatory input for the underlying cell fate decisions. One intriguing example is *Volvox carteri*, a green alga and probably one of the simplest multicellular species. It consists of just two cell types, the small somatic and the large reproductive cells (gonidia). During embryogenesis, *Volvox carteri* undergoes asymmetric divisions, leading to cell size-dependent differentiation (FIGURE 5*C*). Cells bigger than a threshold size become gonidia, whereas the smaller cells differentiate into somatic cells (205). While the molecular mechanism of this size dependence still remains elusive, size-dependent metabolome changes have been proposed to potentially activate cell type-specific gene expression (206).

Size dependency is also observed in the germ-soma differentiation in *C. elegans* (207), where experimentally decreasing cell size disrupts polarization of PAR proteins and the number of asymmetric divisions, altering the germ cell fate program (FIGURE 5*D*). Similarly, during *Arabidopsis thaliana* leaf growth, stem cells of the stomatal lineage (meristemoids) undergo one to five self-renewing asymmetric cell divisions before terminally differentiating into stomatal cells. With each self-renewing asymmetric division, meristemoid cell size decreases (208), and the differentiation into stomatal cells is triggered when a critical cell size threshold is crossed (209). Additionally, cell size-dependent regulation has been speculated to occur during neuroblast differentiation in *C. elegans* (210) and *Drosophila melangaster* (211), where similar to *C. elegans* blastomeres, the neuronal stem cells undergo a fixed number of asymmetric divisions before differentiation.

Moreover, in some plants, the zygote undergoes an asymmetric division, creating two differently sized cells, where the smaller cell becomes the embryo and the larger cell the suspensor, which forms the supporting tissue for the developing embryo (212).

A more detailed overview of how cell size impacts development and differentiation can be found in two excellent recent reviews (213, 214). While the examples highlighted here suggest that cell size is fundamentally linked to developmental processes in multicellular species, future studies are needed to reveal its role in humans.

## 6. CELL SIZE ADAPTATION TO THE ENVIRONMENT

### 6.1. Cell Size Adaptation to Nutrients

In unicellular organisms, nutrient availability is well known to be a major determinant of cell size. Both budding yeast and fission yeast cells get smaller in poor growth media and bigger in rich growth media (51, 215–217). For multicellular organisms, cell size needs to be regulated in a tissue- and cell-type-specific manner. Therefore, cell growth regulation in multicellular organisms is coordinated with nutrient availability as well as growth and proliferation signaling in the form of extracellular growth and mitogenic factors, respectively (172, 218). Thus the effect of nutrients on multicellular organisms may vary from tissue to tissue. The target organism size of the adult animal is set genetically but is sensitive to extracellular factors such as nutrient and growth factor availability during development (172). For example, nutrient deprivation or growth-factor excess during development can lead to size phenotypes in the adult, both at the organismal and cellular level (218), as observed in flies (172, 219). The average height of contemporary European and Central Asian humans has been increasing for the last century, and two strong determinants of this increase were found to be nutrition and genetics (220, 221). While nutrients largely affect human height during childhood and adolescence (222), they can affect human weight at all life stages. For example, when the consumption of a high-caloric, energy-dense diet is combined with reduced energy expenditure, an increase in the size and number of adipocytes is observed, leading to an increase in fat tissue size and body weight (223–225).

Why do cells adapt their size to the nutritional status of the environment? Research into fossil records of fusulinoidean foraminifers has shown that a hyperoxic environment may have enabled gigantism in the single-celled protists around 300 million years ago (226). Atmospheric CO_2_ levels were shown to be strongly correlated with stomatal guard cell size over geological time scales, as observed in plant fossil records (227). A long-term evolution experiment in *Escherichia coli*, spanning 50,000 generations and 32 years, has revealed that the cells continued to evolve larger sizes in rich media, and cell size and fitness remained correlated throughout the 50,000 generations (228). All of the above are examples of size adaptation to nutrient availability on evolutionary timescales. This indicates that size adaptation to nutrients might increase cellular fitness and is therefore selected for.

Therefore, how is nutrient sensing biochemically linked to the corresponding cell size modulation? Nutrients can affect both cell growth (217) and cell cycle progression (229), both of which contribute to the regulation of cell size. This is discussed in detail in the sect. 7.3 on cell size control in changing environments.

### 6.2. Cell Size Adaptation to Physical and Chemical Properties of the Environment

Physical properties of the environment can affect cell size. For example, it has been shown in bacteria that changes in the turgor pressure on the cell wall, which is changes in the difference between the osmolarities of the cell and the extracellular environment, can lead to reversible changes in cell size due to influx or efflux of water (230, 231). Cellular osmolarity-dependent cell volume changes have also been reported for animal and yeast cells (232–236). Cells must sense cell volume changes caused by osmotic perturbations and counteract them to maintain cell membrane integrity and size homeostasis (237). Additionally, external mechanical forces have been shown to affect cell size and cell cycle progression, as discussed in sect. 7.1.5.

An increase in temperature is associated with an increase in enzymatic rates but also an increase in protein denaturation (158, 238). In line with that, for bacteria grown in steady-state conditions, different temperatures affected cell size (239). Cell size changes were also observed in yeast spheroplasts and human leukemia cells facing rapid temperature shifts (240). In all of these cell types, cells were found to be bigger at higher temperatures or after heat shock and this increase in volume could be a reason for heat-shock-induced death (239, 240). A relevant question, therefore, is how global warming might affect cellular size and function in the coming years. Leles and Levine (158) have developed a proteomic model of a phytoplankton cell to study thermal acclimation in phytoplankton. While theoretical studies suggest that cells are expected to become smaller and more heterotrophic with an increase in temperature, their model suggests that under certain environmental conditions, cells might adapt to warming by evolving larger sizes, faster growth rates, and changing their lipid metabolism. Since phytoplankton are responsible for half of all oxygen production on Earth, this finding could be significant for understanding the global effects of ocean warming (158). A study in zebrafish larvae showed cell size-dependent phenotypes under warmer or cooler rearing temperatures, indicating the existence of different selection pressures for ectotherm cell sizes under different temperatures (241).

Similarly, changes in extracellular pH led to changes in cell size in bacteria and yeast (242, 243). In both organisms, acidic extracellular environments led to reduced cell sizes (242, 243), and in bacteria, basic extracellular environments led to longer cells (242). There are also indications that extracellular pH can affect the proliferation of mammalian cells (244, 245). In addition, for cultured mammalian cell lines, the stiffness of the substrate can affect cell size and shape, with a stiffer substrate leading to increased cell volume (246) or area (247). These examples describe how physical properties of the environment can alter cell size and how the cellular response can be either to maintain the original size or to adapt to a size better suited to the environmental change. Moreover, this indicates that multiple environmental parameters signal to a complex cell size control system. The effect that a multicellular environment such as a tissue may have on cell size control is discussed in sect. 7.1.5.

## 7. MECHANISMS OF CELL SIZE CONTROL

### 7.1. Steady-State Cell Size Control in Proliferating Cells

**FIGURE 6.**
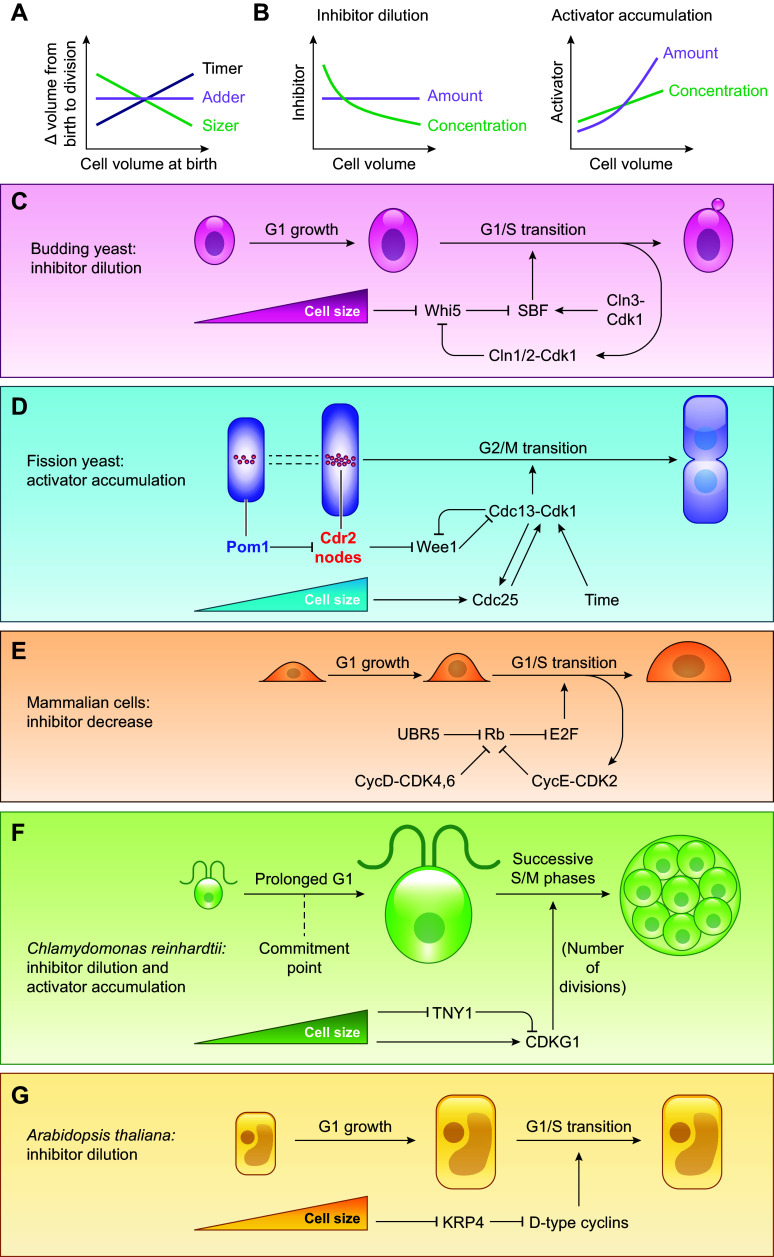
*A*: cell size control of proliferating cells requires that cell cycle progression is coordinated with cell growth. Different types of strategies include sizes, adders, and timers. *B*: on a molecular level, cell size sensing can be implemented through subscaling cell cycle inhibitors or superscaling activators. *C*–*G*: specific cell size reporters have been identified in yeast, mammals, algae, and plants.

Cell size control is the regulation through which cell populations maintain narrow size distributions by correcting deviations in size. Cell size homeostasis then emerges from single-cell level size control, which, in proliferating cells, is typically executed through a coupling of cell division and cell growth to cell size (FIGURE 6). While our understanding of the coupling between growth rate regulation and cell size is only at the beginning (248), the coupling between cell division and cell size has been extensively studied and multiple mathematical models have been proposed. Conceptually, simplified mathematical descriptions have often been categorized into sizers, adders, or timers, depending on what is prioritized in the cellular decision to divide (249–253) (FIGURE 6*A*).

Sizers prioritize attaining a specific cell size at a given point of the cell cycle, for example, at division. In this kind of size control, cells that are bigger at birth grow less during the cell cycle whereas cells that are smaller at birth grow more to achieve similar sizes at division. Adders, on the contrary, add the same volume (or mass) in every cell cycle, independently of birth size. While strong sizers that are close to an ideal sizer mechanism can correct size deviations within one cell cycle, adders require multiple generations. A third kind of size control regime, described as timers, prioritizes keeping cell cycle durations constant. In cells that grow linearly, i.e., cells in which growth rate is constant across cell sizes, both big- and small-born cells would grow the same in a given amount of time. Thus a timer in linearly growing cells has the same emergent phenotype as an adder. For cells that grow exponentially, the growth rate increases proportionally with cell size. In such cells, a timer leads to a continuous broadening of the size distribution over time, and no longer qualifies as a size control mechanism. Timers, in particular for distinct cell cycle phases, are sometimes observed in conjunction with sizers because while timers cannot correct size deviations in exponentially growing cells, they can largely maintain the size homeostasis instated by a sizer.

For size to be controlled by a sizer- or an adder-like mechanism, a molecular signal that measures cell size is required. While the aforementioned size control models have been amply reviewed previously (254), our goal here is to describe the range of mechanisms that facilitate the molecular size sensing required for these models.

One way for a cell to sense its size is by comparison of two biochemical properties: one that changes with cell size (a “reporter” property) and one that stays constant as cell size changes (a “constant” property), similar to a titration. Multiple different size-sensing mechanisms have now been discovered in different species, with this kind of regulation as the underlying strategy. It is important to note here that since the total protein amount in a cell typically scales with cell size, the concentrations of most proteins in a cell size regulation network may be constant and size-independent. Given that, the size scaling of the whole regulatory network may serve as the constant property for size sensing.

#### 7.1.1. Dilution of a reporter.

As described in the section on biosynthetic scaling above, total protein and total mRNA content typically scale with cell size. However, individual proteins and their transcripts may scale differently. Strongly subscaling or superscaling proteins make great candidates for reporters of cell size (FIGURE 6*B*). In the budding yeast *Saccharomyces cerevisiae*, for example, the cell cycle inhibitor protein Whi5, a functional analog of Rb, is subscaling both at the time of cell birth and during G_1_ (4, 50–53), and its reducing concentration serves as a reporter of cell size (FIGURE 6*C*). Whi5 binds to and inhibits the transcription factor SBF [functional analog of E2F (255)], whose target genes drive the G_1_/S transition (256, 257). The number of SBF-binding sites in the genome remains unchanged during G_1_, and the antagonists of Whi5, the G_1_/S activators Cln3 and Bck2, and the SBF-subunit Swi4 are maintained at roughly constant concentrations or are even superscaling (50, 62). Thus, although the exact mechanism of how the decreasing Whi5 concentration is translated into an increased activity of SBF-controlled genes is unclear, both the DNA and the activators can serve as the constant property for the reporter to be titrated against. This mechanism sets up a size-dependent likelihood of commitment to the G_1_/S transition (Start), with bigger cells being more likely to enter the S phase (50, 162). Crucially, all cells have a similar amount of Whi5 at birth. This leads to a higher Whi5 concentration in smaller born cells, keeping them longer in G_1_ and allowing them to grow more. Whi5-dilution-based size-sensing results in an imperfect sizer during budding yeast G_1_, with G_1_ growth being negatively correlated with volume at birth.

A similar mechanism has recently been described in the shoot apical meristem of the flowering plant *Arabidopsis thaliana*. While both the G_1_/S and the G_2_/M transitions are size-dependent in the shoot apical meristem, cell size regulation primarily occurs at the G_1_/S transition (57, 258). D’Ario et al. (57) propose that this G_1_/S size regulation is implemented through the size-based dilution of the KIP-related protein 4 (KRP4), a CYCD/CDKA inhibitor (259), which indirectly inhibits the G_1_/S transition (FIGURE 6*G*). In this scenario, KRP4 serves as a subscaling reporter. Like Whi5 in budding yeast, KRP4 is partitioned equally between sister cells. This is likely due to its association with mitotic chromosomes. Consequently, cells that are born smaller have to grow more to dilute KRP4 enough to proceed through the G_1_/S transition (57).

Animal cells also show birth size-dependent growth during G_1_ (260–264), which is mediated via a similar sizer mechanism: a decrease in the concentration of the cell cycle inhibitor protein Rb as G_1_ progresses (54, 56) (FIGURE 6*E*). Rb inhibits the G_1_/S transition by inhibiting E2F, the main G_1_/S transcription factor in mammals. Rb serves as a subscaling reporter, and the decrease in its concentration during G_1_ is attributed to its degradation via the E3 ligase UBR5 (56). Rb is phosphorylated by CyclinD-Cdk4/6, and at the commitment point, a positive feedback loop involving Cyclin E-Cdk2 leads to Rb hyperphosphorylation, enabling transition into S phase (54, 56, 265).

Moreover, recent work in animal cells has shown that size homeostasis employs not only size-dependent modulation of cell cycle phase durations but also size-dependent modulation of growth rate (252, 264). Specifically, Cadart et al. (252) and Ginzberg et al. (264) showed that cell-to-cell variability and perturbations of cell size or cell cycle length can lead to compensatory changes in growth rates of animal cells. Liu et al. (266) suggest that this size-dependent reduction of growth rates in large cells is executed through the activation of global protein degradation by proteasomes. Additional information can be found in a detailed review by Liu et al. (248).

It should be noted here that size control studies in mammalian cells have largely been performed in cultured cell lines. While the aforementioned sizer is observed in some cell lines (54, 267), immortalized or primary cell lines often exhibit adders over the entire cell cycle (252). In part this can be rationalized by the fact that many of these cultured cell lines carry cancer mutations, which disrupt the G_1_/S regulation (268) and thereby affect size control. In addition, how growing mammalian cells outside of their natural tissue environments affects cell size control can only now be answered thanks to the advent of size control studies performed in intact tissues of living animals (269–271). In vivo mouse epidermis cells exhibit a sizer over the full cell cycle, which arises from Rb-dependent G_1_ size control (270–272). In vivo, cells can have fivefold longer G_1_ than cultured cells, while showing comparable S/G_2_/M lengths (252, 270, 272). Proulx-Giraldeau et al. (272) hypothesize that the emergent size-control behavior over the complete cell cycle depends on the relative lengths of the sizer-G_1_ phase and the timer-S/G2/M phase. Their model shows that the size control mechanism of the entire cell cycle is dominated by the size control mechanism observed in the longer cell cycle phase.

#### 7.1.2. Accumulation of a reporter.

Similar to subscaling inhibitors of cell cycle progression, superscaling activators can also be reporters of cell size. In this case, a protein accumulates with cell growth and its increasing concentration serves as a readout of cell size. Such a strategy for size control has, for example, been observed in the single-celled alga *C. reinhardtii* (47) (FIGURE 6*F*). To understand how accumulation of a reporter is converted into a size signal in *C. reinhardtii*, it is important to first understand its unique multiple fission cell cycle.

Given the alga’s photosynthetic abilities, its cell cycle can synchronize to a diurnal light cycle. If *C. reinhardtii* cells are grown in a cycle of, for example, 12 hours of light followed by 12 hours of darkness, the growth phase (G_1_) occurs during the light phase and the division phase (S/M), also known as the multiple fission phase, occurs during the dark phase (273). Under favorable conditions, *C. reinhardtii* cells can grow more than 10-fold in size in a prolonged G_1_ phase of between 10 to 14 hours (273) and then undergo rapid successive S/M phases, each ∼30 to 40 minutes long, to give rise to multiple daughter cells. Daughter cell size is close to uniform. The number of divisions, and thus the number of daughters born, therefore depends on the growth of the mother cell in the elongated G_1_. This indicates the existence of two size thresholds in the *C. reinhardtii* cell cycle, one that initiates the multiple fission and one that sets the size of daughter cells. The first threshold, also known as the “commitment point,” ensures that G_1_ cells achieve a certain size before entering a division-competent stage (274–276). The second threshold sets the number of rapid division cycles that the committed cell will undergo to achieve a certain daughter cell size. In this manner, both size thresholds are implemented at the G_1_/S transition (276). Heldt et al. (276) propose a model that suggests that both entry into and exit from the multiple fission phase of the cell cycle are controlled by a light-responsive sizer.

In the search for a protein that could facilitate this sizer, an obvious candidate was MAT3, the Rb homolog in *C. reinhardtii*. While Fang et al. (277) showed that a mutation of MAT3 leads to a smaller commitment size and a higher number of divisions postcommitment, Olson et al. (278) showed that MAT3 concentration seems constant as the cell grows in size during G_1_. Another protein implicated in commitment size regulation is the *C. reinhardtii* homolog for CDK1, CDKA1 (279). Epistatic analyses show that both CDKA1 and MAT3 affect commitment via independent pathways (279). The size reporter property for the commitment decision, however, remains elusive. The second threshold, which sets the number of rapid divisions, depends on the accumulating size reporter CDKG1 (47). CDKG1 is a cyclin-dependent kinase that binds D-type cyclins and phosphorylates MAT3. CDKG1 was found to superscale with mother size during late G_1_ and might convey size information to the pathways controlling division (47, 280). Additionally, a recent study (280) has identified a cytoplasmic RNA-binding protein TNY1 that subscales during G_1_ and represses CDKG1, possibly by affecting CDKG1 RNA stability. Hence, the size control system in *C. reinhardtii* seems to employ both dilution and accumulation of reporters as size-sensing strategies.

Another organism where reporter accumulation is clearly employed as a size-sensing strategy is the well-studied rod-shaped fission yeast, *Schizosaccharomyces pombe* (FIGURE 6*D*). Fission yeast size control occurs mostly at the G_2_/M transition and comprises a sizer and a timer that integrate cell length, cell surface area, and time information (60). The fission yeast sizer is one of the strongest size control mechanisms observed in eukaryotes, as it can correct most deviations from mean cell size in a single cell cycle (216).

The G_2_/M transition in fission yeast is initiated by the activation of the cyclin-dependent kinase Cdk1 (281–283) when it is in a complex with the B-type cyclin Cdc13 (284). Cdk1 is phosphorylated and inhibited by the protein kinase Wee1 and is dephosphorylated and activated by phosphatase Cdc25 (281, 285). Thus the balance between Wee1 and Cdc25 tightly regulates Cdk1 activity. Wee1 is in turn inhibited by related kinases Cdr1 and Cdr2 (286–290). Cdr2 is inhibited by the protein kinase Pom1 (283, 285). There are at least three size-dependent signals in the fission yeast cell size control system. Cdr2 forms cortical nodes, which are plasma-membrane-bound multiprotein assemblies concentrated in the central part of the cell, around the nucleus (283, 291, 292). As the cell grows in size, the number of nodes in the central band increases, leading to a higher concentration of nodes. This increase in local Cdr2 node density is coupled to the increase in cellular surface area and integrates cell-surface-area information into the mitotic entry decision (251, 293). The second accumulating reporter is Cdc25. Cdc25 synthesis is coupled to cell volume, leading to higher Cdc25 concentrations in larger cells (58–60, 294). Similar to Cdc25, Cdc13, the fission yeast mitotic B-type cyclin, also superscales with size (59), but Cdc13 concentration has been found to be coupled to time rather than directly to cell size (60). Together, Cdc25, Cdr2, and Cdc13 input cell volume, cell surface area, and time information into the fission yeast size control system, respectively (60). The spatial restriction of Cdr2 nodes is regulated partly by the mitotic inhibitor Pom1, discussed in sect. 7.1.3.

#### 7.1.3. Cell geometry-based gradients.

In this kind of size sensing, the distribution of a reporter with respect to the geometry of a cell changes as the cell grows in size. A well-studied example is that of the fission yeast mitotic inhibitor Pom1, which localizes at the two ends of the rod-shaped cell (283, 285).

Since Pom1 is a cell polarity protein, its concentration is higher at the cell tips and lower in the cell center, forming a spatial gradient in its concentration (283, 285, 295) (FIGURE 6*D*). This spatial gradient consists of stable clusters of Pom1 (296). Cdr2, which is inhibited by Pom1, localizes in cortical nodes in the central part of the cell. Among other factors, Pom1 prevents the Cdr2 nodes from occurring at the cell poles and restricts them to the central band around the nucleus (297, 298). Since the concentration of Pom1 in the center of the cell does not seem to be a major determinant of cell cycle progression in averaged-sized cells, this suggests that Pom1 localization inhibits mitotic entry in very short cells (7, 283, 285, 297).

#### 7.1.4. Overall size control emerges from multiple size control modules.

It is important to differentiate between size control mechanisms controlling individual cell cycle transitions and the resulting size control that emerges over the entire cell cycle. For example, in budding yeast, the G_1_ phase exhibits a weak sizer and the S/G_2_/M phase exhibits a timer, but an approximate adder emerges when the entire cell cycle is considered (162, 253, 272, 299). On the contrary, fission yeast exhibits a more timer-like behavior in G_1_ and a sizer in S/G_2_/M, with the overall cell cycle exhibiting a sizer. Hence, size control may be modular, i.e., cell cycle phase specific (162). Interestingly, a plasticity to fission yeast size control has recently been described, where disrupting one of fission yeast size control’s two size-dependent inputs can shift the size control from an overall sizer to an overall adder (60). This indicates that multiple inputs regulate size control in both fission yeast and budding yeast (299) and the redundancy of these inputs confers robustness and adaptability to size control. That cells have evolved multiple pathways to control size further indicates a high selective pressure associated with an “optimal” cell size.

Perfect sizers, adders, and timers do not occur in nature. For example, a perfect sizer at a cell cycle transition would mean that at a defined threshold size, 100% of cells would make the cell cycle transition and that cells that do not attain this defined size do not transition. In reality, size control is often noisy. In the budding yeast Start sizer, for example, there is no defined size threshold. Cell-to-cell stochasticity leads to cells passing Start at a range of different cell sizes, albeit the likelihood of passing Start increases with an increase in cell size (50, 162). This cell-to-cell stochasticity, coupled with multiple partially redundant pathways contributing to size control, makes identification of molecular size control mechanisms a complex task. One explanation for cells evolving multiple size control pathways could be that each pathway is evolved for a specific environmental condition (300). Size control, so far, has mostly been studied under steady-state conditions, and this could explain why the multiple size control pathways appear redundant. While steady-state studies have been extremely valuable for identifying the various cell size regulators and strategies, studying cell size control under changing environmental conditions may be key to revealing specialized roles for these seemingly redundant regulators.

#### 7.1.5. Mechanosensing in tissues.

Our understanding of size control in vivo is still very limited. One possibility is that in addition to intracellular regulation, tissue context facilitates additional strategies for size sensing and size regulation. Liu et al. (248) outline multiple examples of how extracellular mechanical signals can affect cell size. Kidney epithelial cells have been shown to detect extracellular urine flow via primary cilia and regulate their cell size in response (171). Mammalian cells have been shown to sense local mechanical forces, such as compression or stretching, and to respond by inhibiting or promoting growth respectively (248, 301, 302). In fact, mechanical load is one of the known growth stimuli for human skeletal muscle cells (303). Regular exercise and strength training seem to upregulate mammalian target of rapamycin (mTOR) activity and induce skeletal muscle cell growth (304–306). Recently, a stretch-activated mechanosensor was identified that could link stretch-detection and growth signaling in animal skeletal muscle cells (306) and adipocytes (307, 308). It has long been known that cells stop proliferating when they reach confluence, i.e., fill up the available growth space. This property is known as contact inhibition of proliferation (CIP) (309, 310). Streichan et al. (301) show that the mammalian cell cycle has a mechanosensitive checkpoint at the G_1_-S transition that monitors the space available to the cell before cycle progression. In epithelial cell monolayers, the G_1_/S transition was also found to be sensitive to high mechanical stress, with cells facing higher inter-cellular tension exhibiting a higher likelihood of G_1_/S transition (311). This conversion of a mechanical signal into a biochemical response likely involves proteins of the Hippo-YAP pathway (302, 312–314). Mugahid et al. (315) have shown that YAP controls cell size and cell number via independent circuits. Recently, Stojanovski et al. (314) have proposed a bidirectional coupling between pharynx size and body growth in *C. elegans*, which seems to also be mediated by the mechanotransducer YAP. This suggests a role for mechanosensing in organ size regulation and maintenance of body plan uniformity. Taken together, these observations highlight that to understand cell size regulation of a specific cell type in vivo, it is not sufficient to understand how an isolated cell would sense and control its size, but instead the inputs provided by the surrounding tissue, both mechanical and biochemical, need to be taken into account.

### 7.2. Cell Size Regulation in Nonproliferating Cells

The size control mechanisms described earlier depend on cell division. However, most animal cells in vivo are nonproliferating cells that have temporarily, through quiescence, or permanently, through senescence, exited the cell cycle (316). A consequential question is therefore how size homeostasis is maintained in nonproliferating cells.

On average, proliferating cells double their biomass before division and hence have a high biosynthetic requirement. Nondividing cells are relieved of this replicative biosynthetic burden but surprisingly still exhibit a large range of metabolic activity (161, 172). Lymphocytes, for example, have reduced metabolic rates when quiescent but can upregulate metabolism upon stimulation and conversion to a proliferative and secretory state (317, 318). This kind of a metabolic shift is well-suited to their “waiting and watching” role in the immune response. Quiescent fibroblasts, on the other hand, have protein synthesis rates similar to their proliferative counterparts (318). This could be explained by quiescent fibroblasts being primary synthesizers of the extracellular matrix required for tissue formation and proliferating fibroblasts being important for wound healing after injury (319). These examples debunk the common assumption that nondividing cells are metabolically inactive and instead indicate that they can have a range of biogenic rates depending on their physiological functions (161, 172).

For nondividing cells to be metabolically active and also maintain size homeostasis, the rate of macromolecular accumulation must be balanced by macromolecular degradation and secretion. This would require feedback mechanisms coupling degradation to biosynthesis and maintenance of a fixed level of growth signaling (172). Indeed, studies performed on sensory neurons showed that protein synthesis and protein degradation rates are coupled for long-lived proteins (320). When these neurons were treated with neurotrophin NGF, a growth factor, and increasing amounts of a protein synthesis inhibitor, the degradation rates of long-lived proteins decreased in proportion to the protein synthesis disruption (320). This coupling between degradation and synthesis rates has also been observed at the mRNA level, albeit in proliferating mammalian cells (19). Speculatively speaking, the feedback mechanism between nuclear mRNA concentrations and transcription could also exist in nonproliferating cells. In addition to mRNA transcription being linked to cell size via limiting Pol II (29), it could contribute to mRNA concentrations scaling with size in quiescent cells, at concentrations that are comparable to the concentrations in proliferating cells (16). Another requirement for size homeostasis in nondividing cells is a robust response to osmotic changes in the environment (172). When osmotic challenges lead to changes in cell volume due to influx of efflux of water, a system of osmotic sensors, transducers, and effectors is in place to counteract deformations and restore original cell volume (237). This may be vital for the maintenance of size homeostasis in a dynamic environment.

### 7.3. Cell Size Control in Changing Environments

**FIGURE 7.**
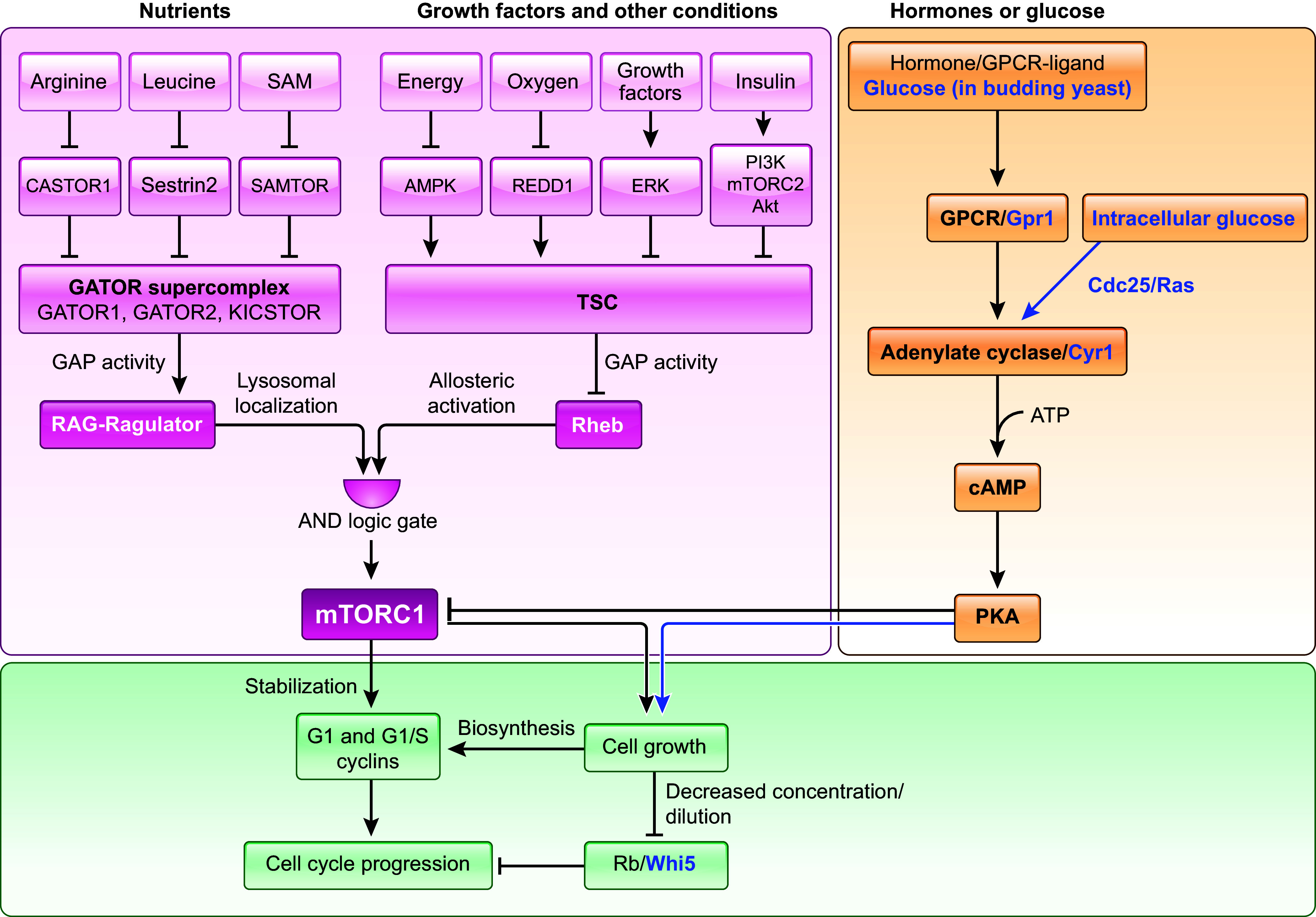
Cell size is adapted in response to environmental stimuli, including nutrient conditions and growth factors. In part, this is achieved through the target of rapamycin (TOR) and PKA signaling pathways, which feed into the regulatory networks controlling cell cycle progression and cell growth. The nutrient-sensing arm of the mammalian target of rapamycin complex 1 (mTORC1) pathway includes the following sensor proteins or protein complexes: the Sestrin2 complex detects cytosolic leucine (326); the CASTOR1 complex detects cytosolic arginine (327); SLC38A9, a lysosomal transmembrane protein, detects lysosomal arginine (328); and SAMTOR detects cytosolic *S*-adenosylmethionine (329). Conserved regulators are shown in black, and budding yeast-specific PKA signaling is shown in blue. GPCR, G protein-coupled transmembrane receptor.

Cell size adaptation to changing environments is implemented by regulation of both cell growth and cell cycle progression. In eukaryotes, this response to nutrient availability, and in the case of metazoans, also to growth factors, is in part attributed to the target of rapamycin (TOR) signaling pathway (321–323). The protein kinase A (PKA) pathway is another signaling pathway found in both mammals and yeast, whose role as a cell growth and cell cycle regulator in response to nutrient availability is well studied in budding yeast (324, 325). Here, we provide a brief overview of how these pathways sense nutrients and regulate cell growth and cell cycle progression accordingly and how they affect each other (FIGURE 7).

#### 7.3.1. Nutrient-sensing and regulation of growth by the PKA signaling pathway.

In budding yeast, the PKA signaling pathway detects the presence of extracellular glucose or sucrose through Gpr1 (330), as well as intracellular glucose via Cdc25/Ras (331, 332). Gpr1, a G protein-coupled transmembrane receptor (GPCR), together with the small GTPase Ras and its guanine nucleotide exchange factor Cdc25 (not to be confused with the phosphatase Cdc25 in mammals, mentioned below) then activates Cyr1, an adenylate cyclase (333, 334). The activated Cyr1 converts ATP to cyclic AMP (cAMP), which serves as a secondary messenger and regulates many physiological processes (335). It activates PKA by binding to its regulatory subunit and releasing its catalytic unit, which in turn triggers phosphorylation cascades downstream of PKA’s multiple targets (335, 336). These signaling cascades mediate the increase in growth associated with the availability of fermentable sugars in the growth media (337). Additionally, multiple cAMP-independent nutrient-signaling circuits for PKA activation have been proposed (338), where the reintroduction of nutrients such as amino acids, ammonium, phosphate, sulfate, iron, and zinc after a period of starvation leads to increased PKA activity via high-affinity transceptors (transporters with an additional receptor function) (339). While the exact molecular mechanisms by which the transceptors activate PKA are still under investigation, it has been shown that some of these transceptors interact with Sch9, a PKA-associated protein kinase (340).

While the core of the PKA pathway is conserved among eukaryotes (335), differences have been observed in its regulation and its upstream activators between species (324, 341). In multicellular organisms, certain cell types, such as pancreatic α- and β-cells and neurons involved in the regulation of the pancreatic endocrine function, are specialized for sensing blood glucose levels (342). Glucose sensing and the subsequent glucagon or insulin secretion by pancreatic α- and β-cells, respectively, are mainly regulated via a network of GLUT2 glucose transporters, ATP-sensitive potassium channels, and voltage-gated calcium channels (343). The role of the PKA pathway in these cells is to enhance or suppress insulin or glucagon secretion by binding to extracellular signaling molecules such as hormones and neurotransmitters (343). Thus, while the budding yeast PKA pathway can be activated by glucose binding, its mammalian counterpart is usually activated by binding hormones or neurotransmitters (324, 344). Additionally, a role for PKA in the regulation of the mTOR pathway has recently been described in mammalian cells. Hormone-sensing GPCRs coupled to a specific type of Gα protein, Gα_s_, can activate PKA, which can in turn inhibit mTORC1, an important complex of the mTOR pathway (345, 346). This interaction could add a layer of hormonal control in cell growth and size regulation.

#### 7.3.2. Nutrient/growth factor-sensing and regulation of growth by mTOR.

The mammalian TOR protein [mTOR; which is also the accepted abbreviation for mechanistic TOR (321)] is a highly conserved protein kinase, which acts as the catalytic subunit of two protein complexes, mammalian TOR complex 1 and 2 (mTORC1 and mTORC2) (332), both of which have been extensively reviewed previously (347–349). The coincidence detector model describes how mTORC1, upon detecting the presence of both nutrients and growth factors, upregulates growth by upregulating ribosome biogenesis, mRNA translation, lipid synthesis, and nucleotide synthesis and downregulating autophagy and lysosome biogenesis (348).

The nutrient-sensing arm of the mTORC1 pathway consists of many dedicated sensor proteins or protein complexes, each specialized to detect the availability of either a particular amino acid or *S*-adenosylmethionine (SAM) by binding to it (347) (FIGURE 7). This nutrient detection is communicated to the mTORC1 pathway by the GATOR supercomplex, comprising the GATOR1, GATOR2, and KICSTOR protein complexes. The GATOR supercomplex alters the nucleotide state of RAG GTPases, which are localized to the lysosome by binding to the lysosome-tethered Ragulator complex (349). RAG GTPases are small guanosine triphosphatases belonging to the Ras superfamily. Upon nutrient detection, the RAG GTPase heterodimers acquire a specific nucleotide configuration, which allows them to promote mTORC1 localization at the lysosome (347, 349). Finally, since the activation of mTORC1 follows the principle of coincidence detection, it functions as a molecular AND gate and needs a second “on” input, for which it relies on the growth-factor-sensing arm of the mTORC1 pathway (347, 349).

The growth-factor-sensing arm of the coincidence detector model relies on the Rheb GTPase and the TSC protein complex. The TSC protein complex can detect multiple stimuli from the environment, such as growth factors, energy, oxygen, and insulin. TSC is localized to the lysosome by tethering to Rheb and upon detection of these stimuli leads to a nucleotide configuration switch in Rheb, making it GTP-bound. GTP-bound Rheb acts as an allosteric activator of mTORC1 kinase activity and provides the second “on” input for the mTORC1 activation AND gate (347, 349).

While the GATOR complexes and RAG GTPases are conserved across most model organisms, conservation of the nutrient-sensing components of the mTORC1 pathway is largely limited to vertebrates (326). This indicates the existence of different nutrient sensors in organisms inhabiting unique environments or prioritizing nutrients different from those important to vertebrates (347). Indeed, *Drosophila* flies were found to have a unique *S*-adenosylmethionine (SAM) sensor that signaled methionine availability to the mTORC1 pathway (350). Thus, while additional sensors remain to be discovered in other organisms, it seems that modular composition of the mTORC1 pathway allows for evolutionarily flexible nutrient-sensing modules to signal to the conserved core of the pathway (350).

The mTORC2 complex is also relevant to nutrient sensing as it plays an important role in glucose metabolism (348). When insulin binds its receptor at the plasma membrane, it triggers a cascade of activation of phosphoinositide 3-kinase (PI3K), mTORC2, and Akt (protein kinase-B) (348, 351, 352). PI3K coordinates glucose intake and utilization (351), while Akt promotes cell growth, proliferation, and survival by inhibiting a range of substrates (348). One of these substrates is the TSC complex, the aforementioned inhibitor of mTORC1 (348). In this manner, insulin detection can lead to activation of the mTORC1 pathway via the mTORC2 complex.

The role of the mTOR network in cell size control is evident from cell size studies performed under mTOR disruption. Inactivating mutations of the *Drosophila* mTOR homolog led to a reduced cell size (353). Genetic analyses linked this cell size phenotype to the S6K kinase downstream of mTORC1. An inactivating mutation of the *Drosophila* S6K indeed led to a small cell and body size (354). In mammals, disruption of the mTOR kinase by rapamycin or of the PI3K kinase (of the PI3K/Akt/mTORC1 circuit) by the drug LY294002 led to a decreased growth rate and a smaller cell size (355, 356). In budding yeast, Kellogg and coworkers (357–359) proposed that TORC2 is involved in the nutrient-dependent regulation of cell size and cell growth, in a manner that is dependent on ceramides.

#### 7.3.3. Regulation of cell cycle progression by mTOR and PKA.

Apart from regulating growth, the nutrient and growth-factor sensing module of the mTOR network also directly affects cell cycle progression. The phosphorylation cascade initiated by the binding of growth factors to their receptors leads to increased transcription of cell cycle genes, such as G_1_ cyclins and cyclin-dependent kinases (CDKs) (323). In addition, growth factor signaling stabilizes G_1_ cyclins by protecting them from protein degradation (323), thereby promoting G_1_/S transition in the presence of nutrients and growth factors.

**FIGURE 8.**
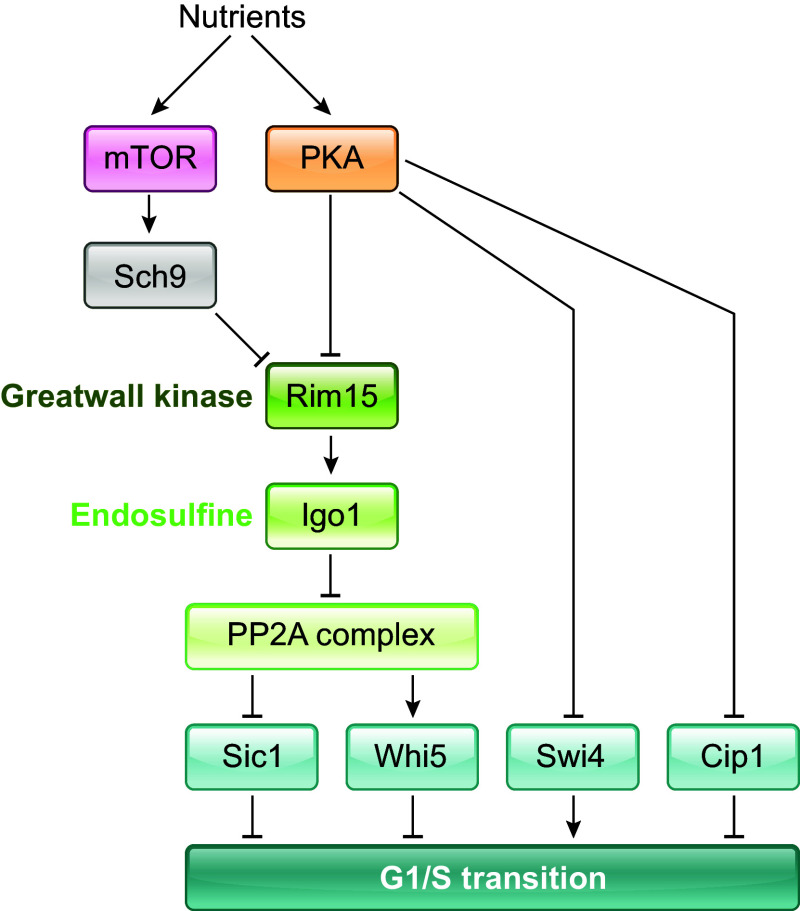
In budding yeast, the Greatwall kinase Rim15 is one major regulator of nutrient-dependent cell-cycle progression. In the presence of nutrients, mammalian target of rapamycin (TOR) and PKA signaling both inhibit Rim15. Through activation of the downstream phosphatase PP2A and its targets Sic1 and Whi5, this regulates the G_1_/S transition.

In budding and fission yeasts, cell size adaptation to changing nutrients can be partly explained by the regulation of the Greatwall-Endosulfine pathway by mTOR and, in the case of budding yeast, also by PKA (360, 361) (FIGURE 8). The Greatwall-Endosulfine pathway is highly conserved from yeast to mammals and serves as a molecular switch to aid cell cycle transitions (361, 362). The Greatwall kinase Gwl (also known as Rim15 in budding yeast, and Ppk18 and Cek1 in fission yeast) promotes phosphorylation of Endosulfine, made up of the two small proteins named ENSA and ARPP-19 in animal cells, Igo1 and Igo2 in budding yeast, and Igo1 in fission yeast (363). Phosphorylated Endosulfine potently inhibits PP2A/B55, the protein phosphatase 2 A subcomplex bound to its regulatory subunit B55. When active, the PP2A phosphatase dephosphorylates CDK/Cyclin substrates, activates CDK/Cyclin inhibitors such as Wee1, and inhibits CDK/Cyclin activators such as Cdc25 (360, 364, 365). Thus active PP2A antagonizes CDK/Cyclin activity and thereby prevents cell cycle transitions (361).

The availability of nutrients leads to an upregulation of TORC1, which leads to an activation of the conserved TORC1 target Sch9 kinase in budding yeast (Sck2 in fission yeast) (360, 366). The Sch9 kinase phosphorylates and inhibits Rim15, the budding yeast homolog of Greatwall, thereby preventing cell cycle progression in nutrient-rich conditions and promoting larger cell sizes (361, 367). This nutrient-based regulation of the cell cycle by the TORC1-Greatwall-Endosulfine-PP2A circuit takes place at the G_1_/S transition in budding yeast and at the G_2_/M transition in fission yeast. PP2A regulates G_1_/S transition inhibitors Whi5 and Sic1 in budding yeast (368, 369), and G_2_/M regulators CyclinB/CDK1, Cdc25, and Wee1 in fission yeast (360, 361).

In budding yeast, the PKA pathway also regulates the G_1_/S transition in response to nutrient availability by inhibiting the Greatwall kinase Rim15 (370, 371) [see Ewald (325) for a detailed review]. In rich nutrients, when both TOR and PKA are active, Rim15 is inactive, promoting PP2A activity. PP2A in turn maintains active Whi5, leading to low CDK activity and ultimately allows progression through Start, the commitment point of the G_1_/S transition, only at larger sizes. Paradoxically, PP2A also promotes S-phase entry by facilitating the degradation of the Cyclin/CDK inhibitor Sic1. Additionally, the PKA pathway affects the G_1_/S transition independently of the Greatwall-Endosulfine network. PKA inhibits Swi4, a component of the major G_1_/S transcription factors SBF and MBF (372). In line with the regulation through Greatwall, this therefore delays Start in rich nutrients, promoting larger cell sizes. However, in rich nutrients, PKA also inhibits the expression of Cip1, an inhibitor of the Cyclin/CDK complex, leading to high CDK activity and a higher likelihood of cell cycle progression (373, 374).

In summary, evidence from budding and fission yeasts indicates that mTOR and PKA regulate cell growth and cell cycle progression in a nutrient-dependent manner. Since the components of mTOR, PKA, and Greatwall-Endosulfine pathways are well conserved, it is possible that these pathways mediate nutritional control of cell cycle progression in multicellular organisms as well. Another fundamental but poorly understood way how nutrients can modulate cell cycle regulation is through the cell cycle-dependent expression of many cell cycle regulators. Since nutrients affect translation rates as well as the relative durations of cell cycle phases, this will inevitably lead to nutrient-dependent regulation of the concentrations of those cell cycle regulators, feeding back on cell size. Indeed, it has been reported that nutrient-dependent changes in cell cycle duration modulate Whi5 concentrations and thereby cell size in budding yeast (51).

## 8. PATHOPHYSIOLOGICAL CONSEQUENCES OF ALTERED CELL SIZE

Given that cell size impacts many of the major cellular functions, including cell growth, cell cycle progression, protein homeostasis, and organelle function, it may not be surprising that misregulation of cell size has been associated with various human diseases (7). For example, increased adipocyte size is associated with obesity and type 2 diabetes (225), α-motoneuron degeneration in amyotrophic lateral sclerosis (ALS) is correlated with their size (375), hypertrophy of cardiomyocytes is associated with increased risk of cardiac disorders (376, 377), and cancer cells often exhibit unusually heterogeneous size distributions (7). The latter may be attributed to the fact that a hallmark of cancers is misregulation of cell cycle and growth, and cancer cells often carry mutations in cell cycle regulators that are known to be involved in cell size regulation.

In addition to increased cell size, small cell size has also been associated with several diseases. For example, neurological diseases such as Alzheimer’s (378), autism (379), and schizophrenia (380) have been correlated with cellular atrophy of neurons as well as the supporting glial cells. Moreover, for muscular atrophy (381) and glaucoma in monkeys (382), a decrease in size has been observed for myocytes and relay neurons, respectively. In some cases of iron deficiency anemia, the red blood cells exhibit a smaller size than usual, a condition known as microcytic hypochromic anemia (383).

**FIGURE 9.**
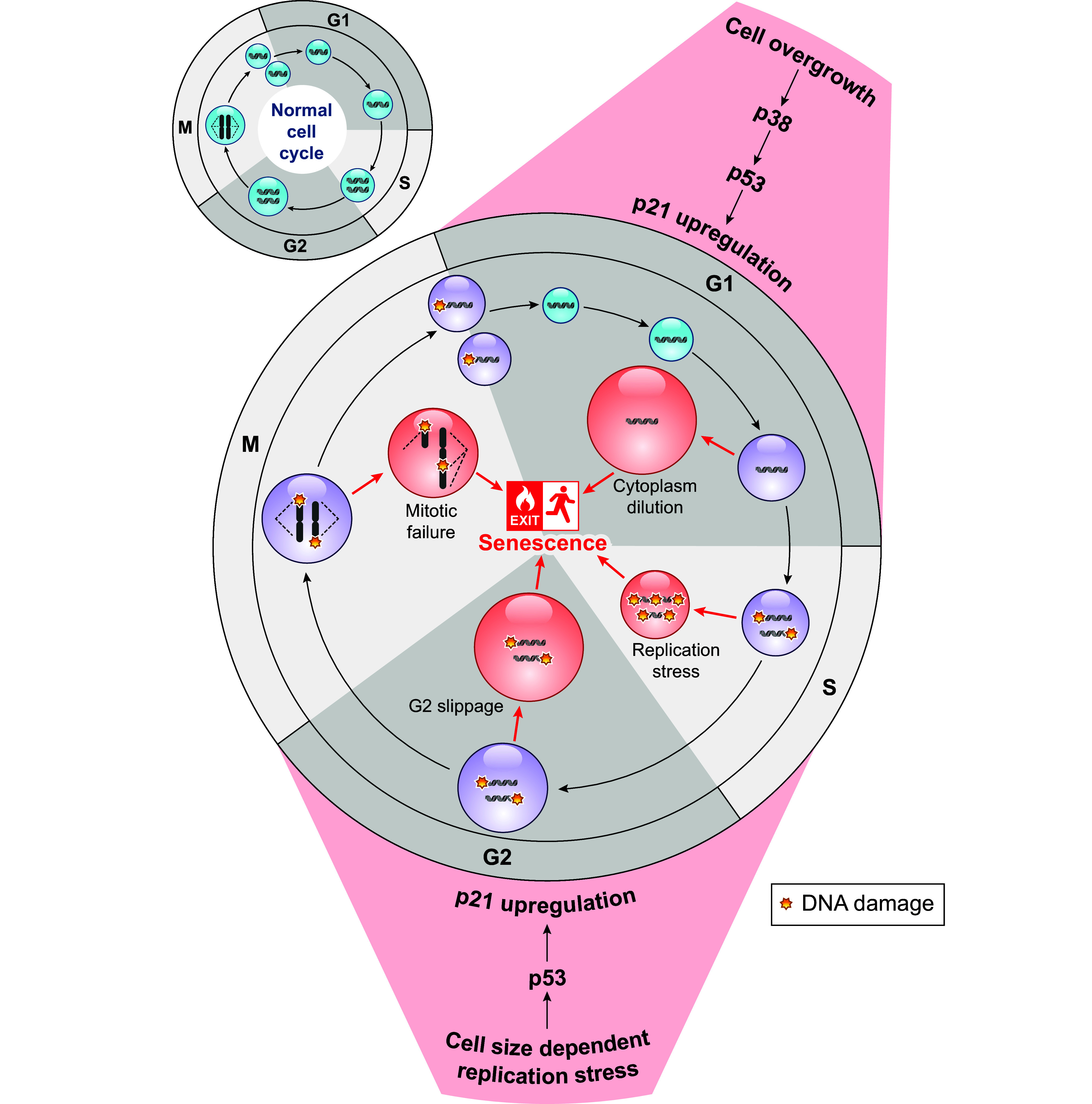
Large cell size promotes cellular senescence through cellular malfunction at different stages of the cell cycle. In G_1_, excessively increased cell size leads to cytoplasm dilution, which promotes permanent cell cycle exit. If large cells enter the cell cycle, DNA damage and impaired DNA repair cause cell cycle exit and mitotic failure.

Despite common observations that altered cell sizes are correlated with disease states, it is often difficult to dissect the causal relationships. For example, large cell size has long been recognized as a hallmark of cellular senescence and aging. However, it has long not been clear whether this reflects a contribution of cell size to cellular malfunction or whether the increased cell size is a byproduct of a decreased cell division rate. Only recently, it was shown that an enlarged cell size can be a driver of cellular senescence and aging (8–10, 384) (FIGURE 9).

### 8.1. Cellular Senescence

While it was long thought that the large size of senescent cells is a consequence of continued cell growth despite their exit from the cell cycle, it was already suggested about 15 years ago that large cell size itself promotes senescence (385). A landmark study by Neurohr et al. (3) then used budding yeast as a model to study the mechanistic consequences of drastic cell enlargement on cell function. They found that large cell size impairs gene expression and cell cycle progression and attributed this cellular malfunction to a decrease in mRNA and protein concentrations in large cells. While within a range around the optimal cell size, protein biosynthesis roughly scales in proportion to cell size (sect. 2), at very large cell sizes the nuclear DNA becomes limiting for transcription, and as a consequence eventually also for protein synthesis. In mammalian cells, large cell size not only leads to a global dilution of the cytoplasm (3) but also to a remodeling of the cellular proteome that resembles the changes usually associated with senescence (5, 46).

A valuable strategy to investigate the consequence of increased cell size in mammalian cells has been a temporary treatment with drugs that induce a G_1_ arrest during which cells keep growing (3, 5). Comparison with a control experiment in which cell growth is reduced, for example, through a simultaneous treatment with rapamycin or a reduction of the serum concentration, then allows to disentangle the causal contribution of increased cell size and the cell cycle arrest itself. Using this approach, several recent studies identified multiple pathways through which strongly increased cell size leads to cell cycle exit (3, 5, 386–389) (FIGURE 9). First, p53- and p38-dependent p21 upregulation in large G_1_ cells, potentially due to an osmotic stress response, can prevent cell cycle entry (5, 388, 389). Second, enlarged cells that manage to enter the S phase may experience replication stress. Due to impaired DNA damage repair, this then leads to mitotic failure and permanent cell cycle exit, either from the G_2_ and M phases or from the next cell cycle (388–390). For more details on the link between cell size and senescence, we recommend a recent review by Manohar and Neurohr (9).

### 8.2. Implications for Cancer Treatment

The observation that excessive cell growth during a prolonged G_1_ arrest, as caused by CDK inhibitors such as the cancer drugs palbociclib and samuraciclib, leads to cellular senescence may also have important implications for cancer treatment (386, 387). Since it is the increased cell size caused by the G_1_ arrest, rather than the arrest itself, which causes permanent cell cycle exit, the fast cell growth of tumor cells seems to be a key requirement for their selective sensitivity to drug treatment. Indeed, oncogenic mutations affect the probability of cells to enter senescence in vitro (386, 387). Obtaining a better understanding of how cell size determines drug-induced senescence in vivo will therefore provide important insights for patient stratification and multidrug treatments. In particular, the dependence on cell size needs to be considered when pairing CDK inhibitors with drugs that modulate cell growth.

### 8.3. Aging

In addition to senescence, enlarged cell size recently has also been causally linked to aging, both in yeast (3) and mammals (11). For yeast, it has long been clear that because mother cells grow between each budding event, replicative aging leads to a continuous increase in cell size. More recently, it has been reported that the replicative lifespan of budding yeast, that is the number of budding events a single cell undergoes before exiting the cell cycle, depends on the initial size of the cell: If young cells are larger, either due to a temporary G_1_ arrest (3, 391) or due to cell size mutations (392), they will undergo fewer divisions. Lengefeld et al. (11) showed that increased cell size also contributes to aging in mammals. Specifically, they found that the size of hematopoietic stem cells increases with the age of mice and humans, which causes decreased proliferation and thus stem cell potential. The recent mechanistic work linking cell size to cellular senescence described above also sheds light on why increased stem cell size can contribute to aging. However, this leaves the question of why older stem cells would be bigger to begin with? One intriguing explanation is that stress conditions, in particular DNA damage, typically lead to cell cycle delays. Because cells still keep growing despite the cell cycle arrest, this leads to increased sizes. Above a certain threshold, the impaired function of larger cells then causes problems in the next cell cycle, leading to a vicious cycle that eventually leads to excessively large cells and permanent cell cycle exit [see Davies et al. (8) for a dedicated review].

Telomere shortening (393) and cell size have both individually been linked to senescence and aging. Moreover, across individuals, shorter telomeres are correlated with larger mean red blood cell size, even after accounting for age, suggesting that telomere length and cell size might be causally linked (394). Indeed, inducing shortened telomeres through reduced telomerase activity leads to DNA damage and increased cell size (395). On the other hand, no dependence of telomere length on cell size has been found in cell size-sorted primary human lung fibroblast (5), suggesting that increased cell size itself does not cause telomere shortening.

### 8.4. Adipocyte Size and Type 2 Diabetes

Adipocytes are a major component of adipose tissue and essential for its function. The size of adipocytes is highly heterogeneous and adapts to external inputs. To store excess energy, adipose tissue expands by an increase in both cell number and cell size. The size of adipocytes is a crucial determinant of metabolic function and has been linked to type 2 diabetes (225, 396). In particular, in the context of severe obesity, increased adipocyte size predicts insulin resistance, even after accounting for total fat mass (397, 398). However, the underlying mechanisms remain largely unclear.

## 9. CONCLUDING REMARKS

**FIGURE 10.**
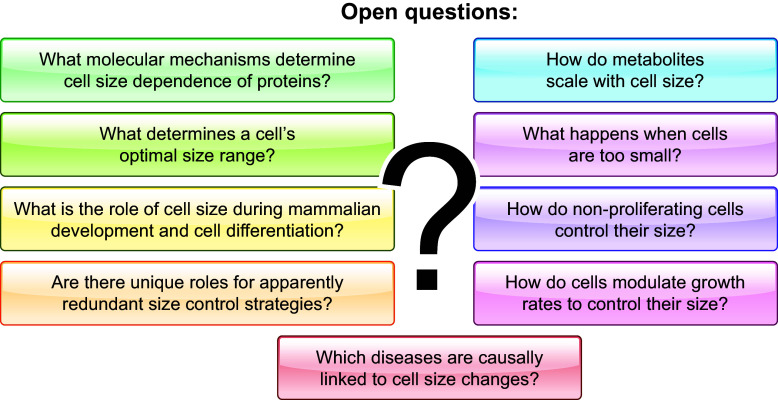
Open questions in the field of cell size control.

For a long time, cell size, in particular the question of how cells control their own size, has been studied as a mostly isolated problem in the cell cycle community. Driven by the finding that changes in cell size cause a global change of absolute and relative protein concentrations, this situation started to rapidly change in the last few years. In essence, the broad changes across the proteome suggest that cell size has the potential to affect almost any biological process. The causal contribution of cell size to aging and cellular senescence are two prominent examples, but it seems likely that the coming years will bring to light many more cell size-driven phenomena. In any scenario where cell size changes in response to drug treatment, biological processes, or genetic or environmental perturbations, it will be critical to reassess the changes of the transcriptome, proteome, or metabolome that are attributed to the specific process by “normalizing” the purely cell size-dependent signature. An important prerequisite for this will be the careful quantification of these cell size-dependent changes. Using orthogonal strategies of cell size-dependent sorting or cell size manipulation, the first studies have already succeeded in distilling the genome-wide changes caused by changes in cell size. However, at this point, it is not yet entirely clear to what extent these depend on biological context, such as cell type or environmental conditions. Understanding how cell size affects specific cell types will also be of major importance in revealing potential causal links to cell size and the underlying mechanisms for the many diseases where correlations with altered cell size have been observed (FIGURE 10).

## GRANTS

This work was funded by the Deutsche Forschungsgemeinschaft (DFG; German Research Foundation) No. 431480687, Human Frontier Science Program (career development award to K.M.S.), and Helmholtz Gesellschaft.

## DISCLOSURES

No conflicts of interest, financial or otherwise, are declared by the authors.

## AUTHOR CONTRIBUTIONS

Y.C. and A.K. prepared figures; Y.C., A.K., and K.M.S. drafted manuscript; Y.C., A.K., and K.M.S. edited and revised manuscript; Y.C., A.K., and K.M.S. approved final version of manuscript.
